# A Comparison of Semilandmarking Approaches in the Visualisation of Shape Differences

**DOI:** 10.3390/ani13030385

**Published:** 2023-01-23

**Authors:** Wuyang Shui, Antonio Profico, Paul O’Higgins

**Affiliations:** 1 Department of Archaeology, University of York, King’s Manor, York YO1 7EP, UK; 2 Department of Biology, University of Pisa, Via Derna 1, 56126 Pisa, Italy; 3 Department of Archaeology and Hull York Medical School, University of York, York YO10 5DD, UK

**Keywords:** virtual anthropology, sliding semilandmarks, iterative closest points, non-rigid iterative closest points, predicted surface, mesh geometry, visualization

## Abstract

**Simple Summary:**

This study extends previous work that examined the consequences of using different approaches to locating densely matched points (semilandmarks) over surfaces on subsequent estimates of their average shape and shape variation with size (allometric scaling). In that study, it was shown that different approaches yield different semilandmarks and, thus, different estimates of means, scaling, and distributions of surface shapes, although there is a high degree of consistency among some approaches. In this study, we compare the surfaces obtained by warping surfaces to the different estimates of landmark and semilandmark configurations that arose from the previous study. Such surfaces have utility in practical contexts, for example, in visualising analytic results as reference surfaces to use in the clinic to assess anomalies and the effects of treatment, or as the basis for building models for subsequent functional analyses. We show that these surfaces share many similarities but differ in detail. Thus, visualisations of shapes derived using semilandmarks from non-rigid semilandmarking approaches especially are likely to fairly represent surfaces and differences between them but are not identical. The extent to which these differences are important depends on the particular study context and aims.

**Abstract:**

In landmark-based analyses of size and shape variation and covariation among biological structures, regions lacking clearly identifiable homologous landmarks are commonly described by semilandmarks. Different algorithms may be used to apply semilandmarks, but little is known about the consequences of analytical results. Here, we assess how different approaches and semilandmarking densities affect the estimates and visualisations of mean and allometrically scaled surfaces. The performance of three landmark-driven semilandmarking approaches is assessed using two different surface mesh datasets with different degrees of variation and complexity: adult human head and ape cranial surfaces. Surfaces fitted to estimates of the mean and allometrically scaled landmark and semilandmark configurations arising from geometric morphometric analyses of these datasets are compared between semilandmarking approaches and different densities, as well as with those from warping to landmarks alone. We find that estimates of surface mesh shape (i.e., after re-semilandmarking and then re-warping) made with varying numbers of semilandmarks are generally consistent, while the warping of surfaces using landmarks alone yields surfaces that can be quite different to those based on semilandmarks, depending on landmark coverage and choice of template surface for warping. The extent to which these differences are important depends on the particular study context and aims.

## 1. Introduction

Over the last three decades, landmark-based geometric morphometric (GM) methods have been increasingly applied to quantify and compare size and shape variation and covariation [1,2,3,4,5]. Before performing GM analyses, the definition of a suitable configuration of landmarks in relation to the research aim is required [6,7]. A simple landmark configuration might be perfectly adequate to quantify shape differences appropriate to the question at hand. In studies of biological transformations such as growth or evolution, the landmarks define equivalent points that are ‘the same’ in terms of development or evolution (‘this point turns into that point’ is homologous), but the locations of homologous landmarks and their density are limited by the extent to which they can be identified and usually the presence of identifiable anatomical features, as well as the preservation of material and available time for digitization.

In many biological applications, landmarks cannot readily be identified, e.g., over smooth regions such as the human cranial vault or tooth crowns. In an attempt to provide detailed information on such regions, different approaches have been proposed to mark up semilandmarks (or dense point correspondences) among curves or surfaces between landmarks [8,9]. The method of sliding semilandmarks, which locates semilandmarks by minimising the bending energy of thin-plate splines (TPS) or Procrustes distance [10,11,12], is most commonly used in biology. Alternative semilandmarking methods include rigid registration approaches, e.g., the auto3dgm package [13] based on the iterative closest points (ICP) algorithm [14], and non-rigid registration approaches, e.g., non-rigid ICP (NICP) [15,16] and the optical flow algorithm [17], among others. The fundamental task of these semilandmarking approaches is to transfer the semilandmarks from a template surface (e.g., a mean surface) to the target specimen. It is worth noting that semilandmarks rely primarily on mathematical mappings and/or the similarity of topographic features, rather than developmental or evolutionary equivalences based on prior knowledge. 

Recent studies have assessed the performance of different semilandmarking approaches based on principal components (PCs) [10,13,18,19,20], distance matrices [13,21,22], and the geometric deviation between template and transformed meshes [18,23]. These have found that different approaches yield different semilandmark locations and, thus, result in analytical results that differ to some degree. This was further investigated in a prior study [24] that provided the starting point for the present one. The performance of three of the semilandmarking approaches described above was systematically examined. These included the sliding TPS approach outlined above. The second approach employed hybrid rigid registration combining least-squares (LS) [25] and ICP algorithms (LS&ICP). After using the LS algorithm to fit the template landmarks to those of each specimen, the ICP algorithm rigidly refitted the template to the target, minimising the sum of squared distances between landmarks and estimated semilandmarks, found by searching for the nearest points on the target from the registered template semilandmarks. The third approach (TPS&NICP) [26] used TPS to perform an initial non-rigid registration of the template landmarks and surface to specimens, and then the NICP algorithm [15] was applied to further warp the deformed template surface to each specimen as rigidly as possible, optimizing the cost function by assigning an affine transformation to each vertex, rather than an interpolation function as used in TPS, before transferring the semilandmarks from the template to the nearest point of the specimen surfaces. We compared semilandmarking approaches, differences in the locations of semilandmarks, Procrustes distances between landmark and semilandmark configurations, estimates of mean landmark and semilandmark configurations, PCs of configuration shape, and estimates of allometry. 

Because homology is unknown for regions that were semilandmarked, it is not possible to assess how well semilandmarks represent homology; rather, the focus was on comparing the results of analyses based on semilandmarks between and within methods, with increasing semilandmark density. The analyses showed that each semilandmarking approach yields different semilandmarks locations, which result in differences in each of the comparisons [24]. The sliding TPS algorithm and TPS&NICP approach yielded results that are more similar to each other than those based on LS&ICP. Further, we assessed consistency within methods among results obtained using different densities of semilandmarks, finding that sliding TPS and TPS&NICP approaches are most consistent, especially where true landmarks are dense. The extent to which these differences are important depends on the context, the question being addressed, and the purpose of the study, but all semilandmarking approaches estimate homology with error, the extent of which is unknowable. Therefore, all subsequent statistical analyses that aim to describe developmental or evolutionary transformation are subject to that error and should be treated with an appropriate degree of caution [7,24]. 

Geometric morphometric analyses enable the visualisation of statistical findings, generating landmark and semilandmark configurations that represent shapes or forms (sizes and shapes) of interest such as the mean or allometrically scaled configurations. Surfaces or regular grids are often warped to these configurations to aid the visualisation of shape differences and, where applicable, changes. This is most commonly performed using TPS [27,28]. However, the authors of [6] noted that ‘With sliding semilandmarks, their relative positions on equivalent curves, surfaces, etc. are not singly interpretable, but rather should be read as a whole, respecting the fact that the underlying assumption in their construction is one of equivalence of the curve or surface patch as a whole’. This was recently reiterated [3,29]: ‘the coordinates of semilandmarks along the surface are meaningless, and one cannot interpret the position of single semilandmarks, only the surface geometry that all semilandmarks describe together’. Thus, although semilandmarks are treated as landmarks in statistical analyses, ‘errors’ in their locations (or differences using different methods to locate them) influence statistical outcomes, as was demonstrated in the previous study [24]; visualisations and interpretations of differences should ignore their locations and focus on the shape of the curve or surface they describe. 

It is, therefore, of interest to know the extent to which the shapes of surfaces warped to fit semilandmark and landmark configurations varying in semilandmark density and locations (e.g., arising from different approaches to placing them) are consistent. If different densities and approaches yield identical or very similar visualisations, this may be reassuring in certain practical applications. For instance, a mean surface might be used in clinical work to compare measurements taken on a patient with an estimate of the population mean [30] and facial approximation from the skull alone in the realm of forensic science [31]. Additionally, surfaces from GM analyses are used to virtually repair and reconstruct fossil material [32] and build 3D models for functional analyses such as finite element analysis (FEA) [33]. The extent to which such estimated surfaces differ when derived using different semilandmark densities and semilandmarking approaches is unknown, yet it is important in that it may affect subsequent morphometric or functional analyses. This question is addressed in the present study. 

The main purpose of this study is to empirically test two hypotheses using surface scans of human heads and ape crania: that there are no differences in surface mesh shape (the shape of the configuration of surface vertices and the nodes of the surface mesh, rather than the landmark and semilandmark configuration) between estimates derived using different semilandmarking densities and approaches applied to surfaces representing (a) the mean of a sample and (b) allometrically scaled shapes. 

To these ends, a template surface mesh is warped to fit the estimated mean and allometrically scaled mean landmark and semilandmark configurations derived from different semilandmarking densities and approaches, and the resulting surfaces are compared. Additionally, these surfaces are compared with surfaces warped using landmarks alone. Of interest is the extent to which these surfaces differ and how they differ. The focus is on the comparison of the shape of the surface rather than the geometry of the underlying mesh.

## 2. Materials and Methods

### 2.1. Materials

#### 2.1.1. Datasets, Landmarks and Semilandmarks

We used two datasets comprising surface meshes that exhibit varying degrees of complexity: 100 adult human male heads comprising 16 anatomical landmarks from the Liverpool–York Headspace dataset [34,35] and 20 ape crania consisting of 41 anatomical landmarks (5 *Gorilla*, 5 *Hylobates lar*, 5 *Pan troglodytes*, and 5 *Pongo abelii* [36]. We extracted the external surfaces of heads and ape crania to avoid the internal surfaces interfering with the projection of semilandmarks. The 3D meshes were post-processed by smoothing surfaces, removing the irrelative discrete vertices, and repairing self-intersecting triangle meshes. Compared with human heads, the sample of ape crania of different species shows greater size and shape variation and presents more complex surfaces. 

Similar to the previous study [24], the mean surfaces of heads and ape crania were estimated and used as templates (after landmarking and semilandmarking them) for each dataset to yield semilandmarks among every specimen. For the human head, we selected an arbitrary head as the initial template and then used NICP [15] to align all of the human heads based on landmarks and establish dense point correspondences (identify points on the target surface that match each vertex of the template surface). Next, the mean head was estimated by averaging correspondences among heads. For the ape crania, which vary far more in form, an alternative approach was required. The *k*-means clustering algorithm was employed to sample 800 points over a *Gorilla* cranium; then, the sliding TPS approach [10] was used to yield semilandmarks among specimens. Following this, the mean form of the landmark and semilandmark configurations was calculated, and the mean ape cranial surface was estimated by warping the surface mesh of an arbitrary specimen to fit this configuration. This process of making an initial estimate of the mean follows one commonly used to compute semilandmarks, where an arbitrary specimen is used as an initial template to estimate semilandmark coordinates, and the mean of the resulting landmarks and semilandmarks is used to estimate a new mean template by re-warping the original template to them before re-semilandmarking the sample.

**Figure 1:**
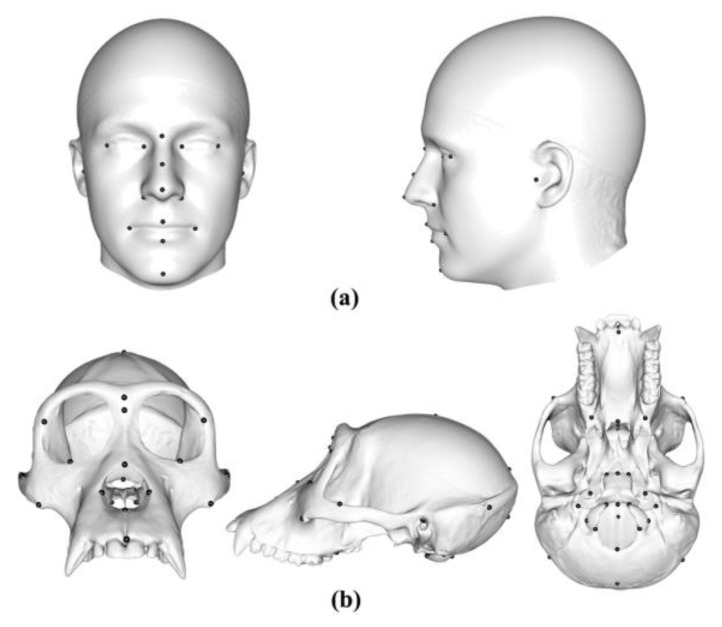
Two datasets. (**a**) The human template head with 16 landmarks. (**b**) The ape template cranium with 41 landmarks.

Figure 1 shows the human template head with 16 landmarks, and Figure 1b shows the ape template cranium with 41 landmarks. Notably, the scalp surface in the headspace data lacks identifiable landmarks, while the ape crania present landmarks over the whole surface. These differences are expected to affect how well semilandmarking is controlled, particularly for sliding TPS, because landmarks are required to control sliding, which is not the case for the other approaches. Sliding TPS was applied over the scalp for the consistency of analyses and comparability of results. 

#### 2.1.2. Semilandmarks

The *k*-means algorithm was used to sample, as evenly as possible, 10 different densities of semilandmarks per square centimetre from the template head: 20 (0.017/${\mathrm{cm}}^{2}$), 40 (0.034/${\mathrm{cm}}^{2}$), 60 (0.052/${\mathrm{cm}}^{2}$), 80 (0.069/${\mathrm{cm}}^{2}$), 100 (0.086/${\mathrm{cm}}^{2}$), 200 (0.172/${\mathrm{cm}}^{2}$), 400 (0.343/${\mathrm{cm}}^{2}$), 600 (0.515/${\mathrm{cm}}^{2}$), 800 (0.688/${\mathrm{cm}}^{2}$), and 1000 (0.858/${\mathrm{cm}}^{2}$) semilandmarks. In addition, the above procedure was repeated to generate five different semilandmark densities among ape crania: 50 (0.129/${\mathrm{cm}}^{2}$), 100 (0.258/${\mathrm{cm}}^{2}$), 200 (0.517/${\mathrm{cm}}^{2}$), 400 (1.033/${\mathrm{cm}}^{2}$), and 800 (2.067/${\mathrm{cm}}^{2}$). Following the creation of the templates, three different semilandmarking approaches were employed to project semilandmarks from the template to every specimen to yield semilandmarks, as follows [24].

(a) Sliding TPS

The sliding TPS approach is the most commonly used approach in biological studies to yield semilandmarks by sliding semilandmarks projected from the template along the tangent direction of a curve or the tangent plane of a surface, minimising the bending energy of TPS [11,37]. In this study, we used the patching (placePatch) and sliding (slider3d) procedures in the *Morpho* R package (version 2.10) to yield sliding semilandmarks at varying densities based on the template [38]. The sliding step minimises bending energy and, thus, depends on landmarks to control the sliding. For the headspace data, no landmarks are present over the scalp, so we expect sliding to be poorly controlled. This situation does not arise with the ape cranial data.

(b) Rigid registration

We used the rigid LS&ICP method to register the template to every specimen based on the fixed landmarks and then projected semilandmarks from the template to each specimen. First, the initial rigid alignment calculated by LS, constrained by landmarks, was performed to fit the template to each specimen. Second, the ICP algorithm rigidly refitted the template to the target, minimising the sum of squared Euclidean distances between landmarks and semilandmarks on the template and specimen. The alignment generated by LS speeds up the convergence of the ICP algorithm. Finally, we projected different densities of semilandmarks from the registered template to each specimen. This was carried out using purpose-built code in the C++ programming language using Microsoft Visual Studio 2015.

(c) Non-rigid registration

We used the non-rigid TPS&NICP method [26] to yield semilandmarks on every specimen. This comprised two steps: First, a triplet of TPS was used to warp the template to every specimen based on the fixed landmarks. Second, the NICP algorithm [15] was applied to warp the deformed template surface to each specimen and establish dense point correspondences based on locally affine regularizations and adjustable stiffness parameters. In this process, preliminary correspondences are established by searching for the nearest points between two surfaces, and then the cost function is optimized. It comprises a landmark term, a local affine regularization term, and a stiffness term and assigns an affine transformation to each vertex. New correspondences are obtained by searching the deformed template surfaces. Registration loops are carried out in which stiffness weights are iteratively decreased and the template is incrementally deformed. This non-rigid method, in contrast to the rigid registration used in LS&ICP, matches the warped template surface closely to each specimen. This was carried out using purpose-built code in the Matlab programming language. 

**Figure 2:**
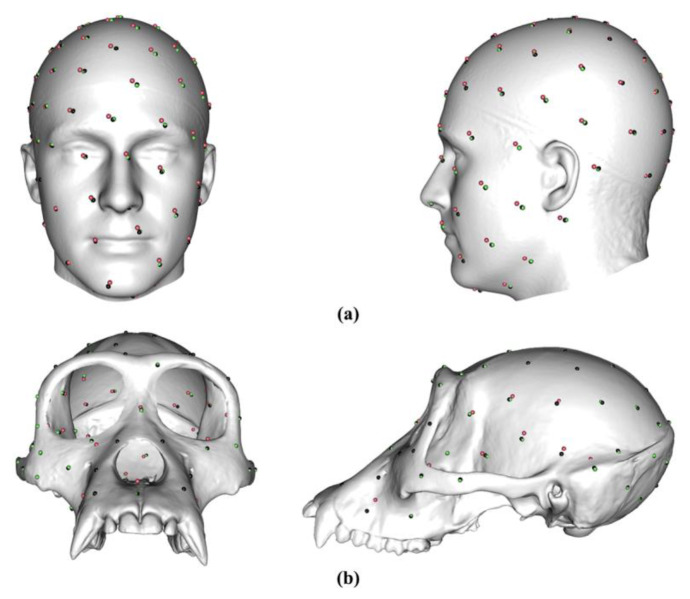
One hundred semilandmarks generated by the sliding TPS (black points), LS&ICP (red points), and TPS&NIPC (green points) approaches. (**a**) Mean adult human head. (**b**) Mean ape cranium.

Figure 2a shows 100 semilandmarks generated by sliding TPS (black points), LS&ICP (red points), and TPS&NICP (green points) on the mean form of the head surface generated by sliding TPS. While semilandmark locations differ between all methods of semilandmarking, the differences are small between sliding TPS and TPS&NICP approaches and a little larger between these and the LS&ICP approach. In contrast, differences are much greater among methods in the ape cranial dataset. Thus, Figure 2b shows 100 semilandmarks on the mean ape cranium generated by sliding. Semilandmarks generated by sliding TPS appear to be in similar locations to those generated by TPS&NICP, but the locations of semilandmarks generated by LS&ICP are quite different.

### 2.2. Methods

#### 2.2.1. Comparisons of Mean Surface Meshes between Different Approaches

**Figure 3:**
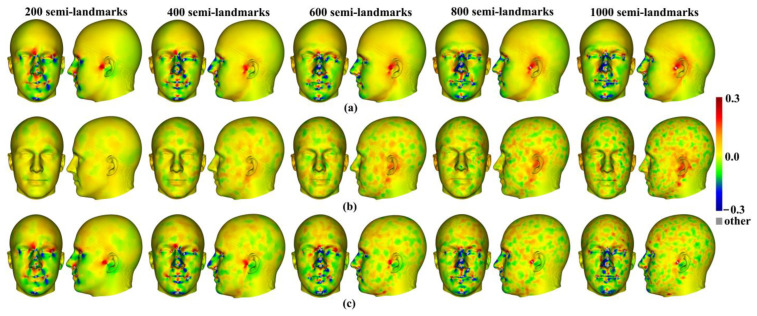
Visualization of the differences in mesh triangle surface areas between mean surface shapes generated using different semilandmarking approaches. (**a**) Differences between sliding TPS (reference) and LS&ICP (target) approaches. (**b**) Differences between sliding TPS (reference) and TPS&NICP (target) approaches. (**c**) Differences between TPS&NICP (reference) and LS&ICP (target) approaches. Scale bar indicates difference in local area between reference and target surfaces expressed as a proportion of the reference area.

For each dataset, we applied GPA to the landmark and semilandmark configurations from each semilandmarking approach and density and then computed the Procrustes mean configurations (centroid size = 1.0). Subsequently, the surface of the template specimen was warped using TPS to fit each mean configuration, thereby generating a ‘mean surface’ consisting of the coordinates of the full set of vertices with identical topology but different relative vertex locations for each estimate of the mean (from each semilandmarking method and density). A vertex is a node of the mesh, and the connections between nodes describe the mesh topology. It should be noted that the template surface was already warped to an estimate of the mean during the semilandmarking process and, as such, under little further deformation in this step. Next, the differences between these estimates of the mean surface shape were quantified and visualized. A hybrid approach was used to quantify global and regional differences in mean surface estimates generated by different semilandmarking approaches. The global comparison used Procrustes superimposition to register mean shape surface mesh vertices generated by different semilandmarking approaches, following which the Procrustes distance between the mean surfaces was calculated, and a principal components analysis (PCA) of mean surfaces was carried out. Additionally, regional differences between estimated mean surfaces were visualized based on (registration independent) colour maps (see example in Figure 3) of surface area differences between each equivalent triangle of the two surface meshes [39]. While these are registration-free depictions of differences in surface area, they incompletely describe the differences between surfaces and should be interpreted in conjunction with the surface renderings of the reference and target shapes. 

However, differences in semilandmark locations and densities between approaches resulted in different mesh vertices locations, even if the shapes of surfaces being compared were identical. This affected visualisations and computations of distances and PCs based on the vertices. 

This is related to the point made by Oxnard and O’Higgins [6], Mitteroecker and Schaefer [3], and Bastir et al. [29] that semilandmark locations on surfaces should not be interpreted singly. In warping the mesh to each semilandmark, the locations of semilandmarks directly control where mesh vertices are located, thus affecting the local geometry of the mesh. Warping transfers differences in individual semilandmark locations to mesh vertices. This is evident from Figure 3, which presents colour maps of differences in mesh triangle areas among mean surface shapes generated using different semilandmarking approaches.

**Figure 4:**
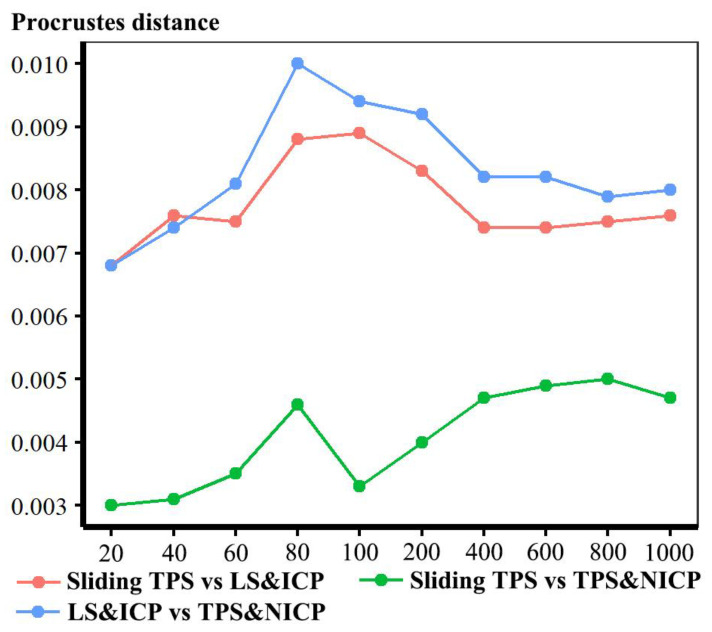
Procrustes distances computed between vertices of the mean surfaces of human heads generated by warping the template mesh to the semilandmarks obtained by different approaches.

In the comparisons in Figure 3, numerous punctate regions of localised differences in areas of triangle meshes are evident, particularly between the sliding TPS and TPS&NICP approaches, where semilandmarks located over the vault are in slightly different places. These lead to the punctate appearance of the colour map. The resulting Procrustes distances between mesh vertices are illustrated in Figure 4. These distances increase between the lowest and highest semilandmarking densities, but this is not directly related to the number of semilandmarks used to warp the meshes. Rather, the figure shows a generally increasing trend but with increases or decreases in Procrustes distance between successive increments of semilandmark density. While some part of these Procrustes distances relates to differences in surface shape, the distances are inflated to an unknown degree by the differences in semilandmark locations over the surface. 

To avoid this problem, a second semilandmarking step followed by a re-warping of the template surface to these new semilandmarks and the landmarks is required on all surfaces to be compared. First, the semilandmarks of the template surface were projected onto the estimated surfaces (e.g., mean or allometrically scaled shapes) generated by different approaches and densities to generate new semilandmarks based on the fixed landmarks (re-semilandmarking). Second, the template surface is warped to fit the original landmarks and new semilandmarks generated by different approaches and densities to produce the surface (re-warping). This eliminates the localised effects on mesh geometry (e.g., more or less deformed triangles within the meshes, while topology remains constant) of differences in semilandmark locations due to the choice of a semilandmarking approach. It focuses the comparison on the shapes of the re-warped surfaces rather than mesh geometry. The sliding TPS and TPS&NICP semilandmarking approaches result in very similar semilandmark locations and consistent statistical results (Figure 2 and [24]). Either could be chosen as the basis for the re-semilandmarking and re-warping of meshes, with little or no effect on the outcome of comparisons. In this study, the sliding TPS approach was chosen because it is most commonly applied in such work. 

The resulting visualisations of differences and Procrustes distances between estimates of the mean surface mesh indicate smaller differences after re-sliding (or re-semilandmarking) and re-warping, as expected. Thus, differences between the mean surface mesh derived by LS&ICP and the other two approaches are relatively large in the face, especially around irregular features such as the nose and mouth, while between sliding TPS and TPS&NICP, the mesh differences are small and diffuse. Procrustes distances generally increase with increasing density, as in Figure 4, but are smaller than those from the original fitting of the template mesh to the semilandmarks from each approach (see Results, Section 3.1.1 for details). 

This re-semilandmarking and re-warping allows mesh surface shapes to be compared between semilandmarking methods. It ignores the local differences in surface mesh triangle areas that will affect the colour maps of differences in the mesh triangle surface areas and refocuses the analysis on the shape of the surface (in the sense of its topography). It was applied to all subsequent comparisons of mean surfaces and allometrically scaled surfaces arising from different semilandmarking approaches in this study. It was also applied to the comparison of surfaces derived with each semilandmarking approach using different densities of semilandmarks.

#### 2.2.2. Comparisons of Allometrically Scaled Surface Meshes

In the previous study [24], the predicted landmark and semilandmark configurations representing the extreme limits (smallest and largest) of the allometric vector, derived using the multivariate regression of shape (the scores of specimens on the full set of PCs) on the natural logarithm of centroid size, were computed using each semilandmarking method and density. This was performed for both datasets. To investigate how differences in semilandmark locations between approaches affect predictions of allometrically scaled surfaces, the template surface was warped to these configurations. Next, as for the comparisons of mean surfaces, these surfaces were re-semilandmarked and re-warped to yield surface meshes before calculating Procrustes distances between mesh vertices, PCs, and visualisations of differences in mesh triangle areas.

#### 2.2.3. Comparisons of Surface Meshes Resulting from Different Semilandmarking Densities

Previous analyses focused on differences in surface mesh predictions arising from the use of different semilandmarking approaches. Further analyses were directed towards assessing the extent to which predicted surfaces differ when produced by each semilandmarking approach using different densities of semilandmarks. This was applied to both datasets. As for the comparisons of mean and allometrically scaled surfaces, the surfaces produced by each semilandmarking density using each semilandmarking approach were re-semilandmarked and re-warped, and then GPA and PCA were carried based on the vertices of the surfaces generated by different densities of semilandmarks from each approach. Procrustes distances and PCAs were used to assess overall shape differences. Colour map visualisations of differences in mesh areas were also produced, but these first required the scaling of the meshes. Because the number of semilandmarks varies, the centroid sizes of the full set of vertices of the surfaces fitted to each mean semilandmark and landmark configuration are inversely related to the density of semilandmarks; i.e., surfaces generated using low densities of semilandmarks are larger than those using high densities. Therefore, to visualise differences in predicted surface mesh triangle areas, the surfaces (configuration of the full set of vertices) were scaled to the same centroid size. 

#### 2.2.4. Comparisons of Mean and Allometrically Scaled Surface Meshes Resulting from Landmarks Alone

In order to assess what, if anything, is gained by using landmarks and semilandmarks to compute mean and allometrically scaled surfaces, the surfaces from the analyses described above were compared with warped surfaces derived using only the landmarks by computing Procrustes distances between the vertices of the template surface mesh warped to fit the mean landmarks or allometrically scaled landmarks from each dataset. The differences between these surfaces and those derived using landmarks and semilandmarks were visualized using colour maps, as described above. 

The template mesh for each dataset is an initial estimate of the average surface, so it is expected that fitting it to the mean landmarks will yield a surface not very dissimilar to the mean surfaces estimated using landmarks and semilandmarks. In practice, it is common to use the surface of an individual close to the mean for visualisation as the template, yet the effects of the choice of template surface are unclear. Therefore, surfaces derived using landmarks and semilandmarks were compared with those derived using landmarks alone, this time using the head surface with the smallest Procrustes distance to the mean (based on landmarks and the maximum number of semilandmarks) and the ape cranial surface used to generate the template cranium. The resulting predictions of mean and allometrically scaled surfaces were compared with those based on the template surfaces. 

## 3. Results

The effects of different semilandmarking approaches and densities on estimates of the mean and allometrically scaled surfaces of human heads were assessed after the surfaces were re-semilandmarked and re-warped, and then, key analyses were repeated using the ape cranial surfaces to compare the performance of approaches on surfaces that exhibit a greater degree of variation and complexity in surface size and shape. Additionally, these surfaces were compared with those warped to fit the landmark configurations (without semilandmarks). 

### 3.1. Comparison of Estimates of Mean Surfaces

The differences in shape of the estimated mean surfaces generated by (1) different semilandmarking approaches and (2) densities are quantified. All of these comparisons and those of allometrically scaled surfaces are based on surfaces derived by re-semilandmarking and re-warping, as described in the methods section.

#### 3.1.1. Different Semilandmarking Approaches

**Figure 5:**
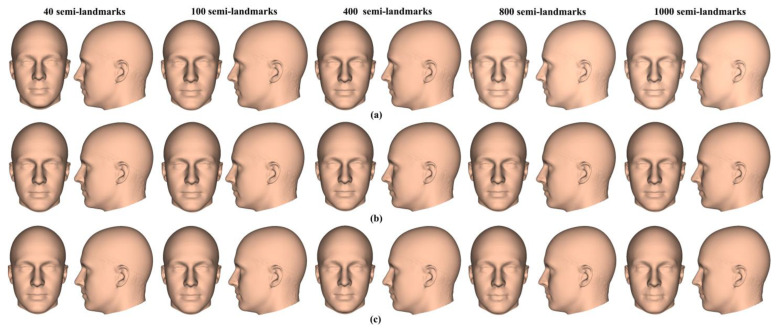
Visualization of the head mesh surfaces generated using different semilandmarking approaches after re-semilandmarking and re-warping. (**a**) Sliding TPS. (**b**) TPS&NICP approach. (**c**) LS&ICP approach. Increasing semilandmark density from left to right.

The mean head surfaces from each semilandmarking approach derived using varying numbers of semilandmarks, after re-semilandmarking and re-warping, are shown in Figure 5. The surface mesh renderings before re-semilandmarking and re-warping are not noticeably different in shape and so are not shown. 

**Figure 6:**
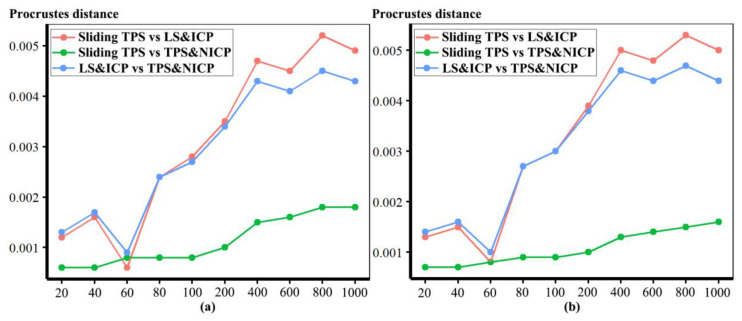
Procrustes distances computed between the mean surfaces of human heads obtained by different approaches after re-semilandmarking and re-warping the template mesh. (**a**) Procrustes distances computed between all the vertices (**b**) Procrustes distances between mean landmarks and semilandmarks.

In Figure 5, all head surfaces after re-semilandmarking and re-warping appear very similar. The main differences are in the detail of the complex regions of the surfaces, where those from LS&ICP appear less sharp, especially around the eyes and mouth. In order to compare these in detail, Procrustes distances were computed between the coordinates of all vertices of the surface meshes of the mean human head surfaces estimated using different semilandmarking approaches and densities (Figure 6a). For comparison, for the same surfaces, the Procrustes distances were also computed between the mean landmarks and semilandmarks (Figure 6b). These distances are very similar, indicating that the re-warping of meshes preserves differences between the landmark and semilandmark sets. In contrast, the re-warping has a marked effect on the Procrustes distances between meshes compared with those warped to the original landmark and semilandmark configurations (see Methods; Figure 4 vs. Figure 6a). 

Comparing these distances between different semilandmarking approaches (Figure 6a) indicates that the full set of vertices of the mean surface generated from sliding TPS are, in general, most similar (smallest Procrustes distances) to those from TPS&NICP at all semilandmarking densities, and these distances increase with increasing semilandmark density. The Procrustes distances between mean surfaces based on semilandmarks from LS&ICP and both sliding TPS and TPS&NICP are, in general, larger and also tend to increase with increasing semilandmark density. 

**Figure 7:**
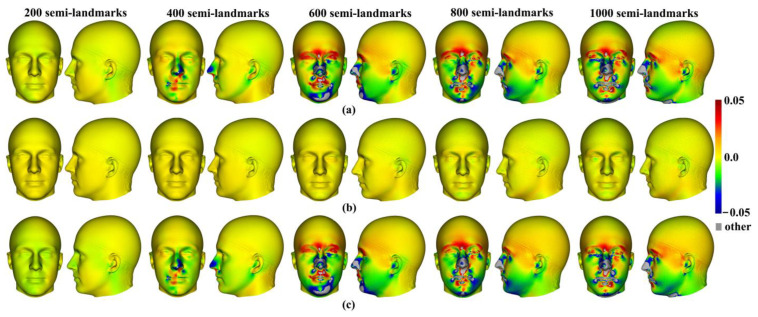
Visualization of the differences in mesh triangle surface areas among mean surface shapes generated using different semilandmarking approaches after re-semilandmarking and re-warping. Differences between (**a**) sliding TPS (reference) and LS&ICP (target) approaches. (**b**) Sliding TPS (reference) and TPS&NICP (target) approaches. (**c**) TPS&NICP (reference) and LS&ICP (target) approaches. Scale bar indicates difference in local area between reference and target surfaces expressed as a proportion of the reference area.

Differences between the mean surfaces of human heads derived from different semilandmarking approaches and densities of semilandmarks are illustrated in Figure 7. This visualises differences in areas of equivalent triangles in the template surface mesh derived from each semilandmarking approach and density after re-semilandmarking and re-warping (see Figure 3). Figure 7a visualises the differences in shape among mean surface meshes from sliding TPS (reference) and LS&ICP. 

Differences in local surface areas between sliding TPS and TPS&NICP (Figure 7b) are very small at all semilandmark densities. The scalp region smoothly presents slightly smaller local surface areas (~ratio of difference in area ~0.01 = 1%; light green) from TPS&NICP relative to sliding TPS. In comparisons between LS&ICP and the other semilandmarking approaches (Figure 7a,c), differences increase markedly with an increasing semilandmark number and are mostly found in the face in regions of complex topography, e.g., the eyes, nose, mouth, and chin, and in which semilandmarks are closer to fixed landmarks. They are much less marked over the scalp. These visualisations reflect the Procrustes distances between surfaces presented in Figure 6a. 

**Figure 8:**
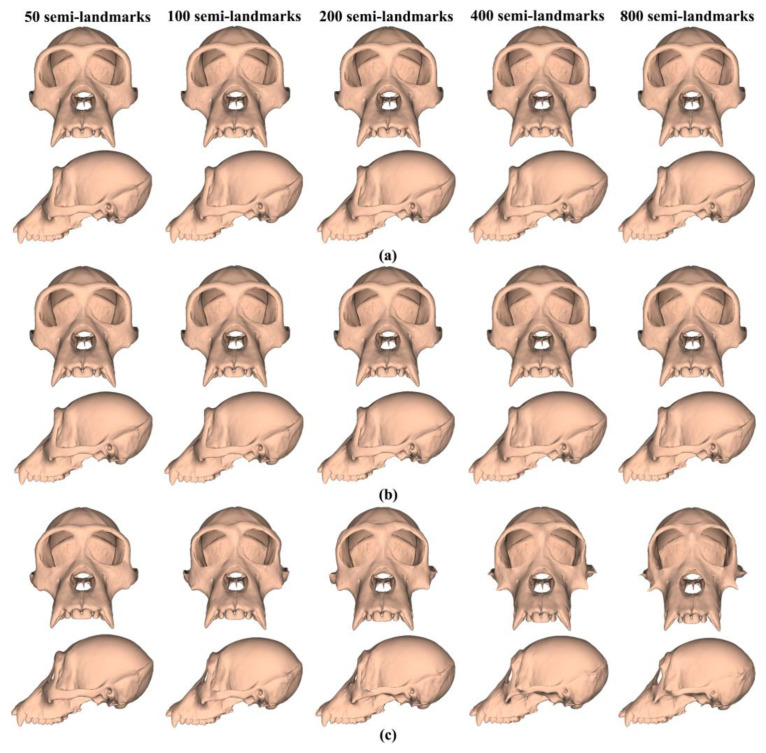
Surface meshes of the estimated mean ape cranium generated using different semilandmarking approaches after re-semilandmarking and re-warping. (**a**) Sliding TPS. (**b**) TPS&NICP. (**c**) LS&ICP.

The analyses described above were repeated with the ape cranial surfaces generated using mean landmarks and semilandmarks. Figure 8 presents the mean surfaces estimated by each semilandmarking approach at varying densities of semilandmarking. As with the headspace data, they appear very similar to the naked eye, with those from LS&ICP appearing slightly different (e.g., zygomatic region) from those derived by sliding TPS and TPS&NICP, especially at higher semilandmarking densities. 

Because LS&ICP yields unreasonable semilandmarks among ape crania (red points in Figure 2b) and results in distinctive estimates of mean ape cranial shape, especially at higher densities of semilandmarking (Figure 8c), we focus on comparing the mean surfaces based on semilandmarks of varying density from sliding TPS and TPS&NICP. Procrustes distances between the coordinates of all vertices of the surface mesh of ape crania warped to the mean landmark and semilandmark configurations are presented in Table 1. These indicate that differences between the full sets of vertices of the mean surfaces generated from sliding TPS and TPS&NICP become greater with increasing density. As with the headspace data (Figure 6a,b), Procrustes distances based on the mean landmarks and semilandmarks of ape crania (Table 1) are similar to those based on the vertices of the surface meshes warped to fit them (Table 2), and the Pearson correlation between these vectors of distances is 0.9940. 

**Figure 9:**
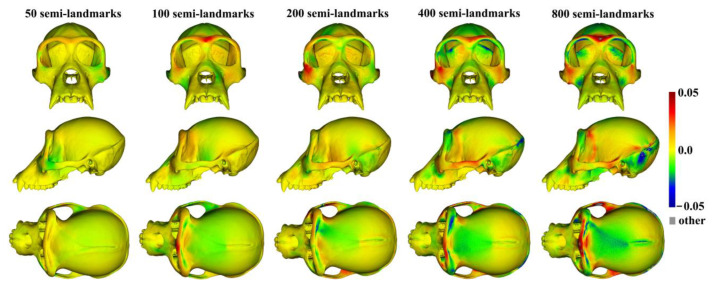
Visualization of the regional differences in local surface areas of mean ape cranial shapes from sliding TPS (reference) and TPS&NICP (target) approaches. Scale bar indicates difference in local area between reference and target surfaces expressed as a proportion of the reference area.

The regional differences between the mean surfaces of ape crania derived from the sliding TPS (reference surface) and TPS&NICP approaches are illustrated in Figure 9. This figure reflects the Procrustes distances of Table 1 in indicating that differences in mean surfaces become greater with increasing semilandmarking density. The differences are concentrated in the vicinity of more complex surface regions, e.g., sagittal crests, supraorbital ridges, the zygomatic arch, the temporal fossa, and the nuchal crest. 

#### 3.1.2. Different Densities of Semilandmarks

**Figure 10:**
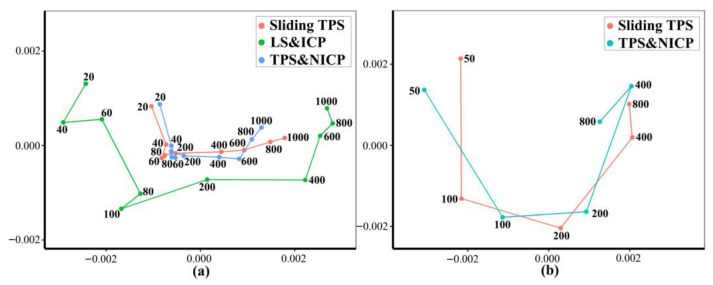
Superimposed scatterplots of PC 1 and PC 2 of mean shape surfaces using sliding TPS (red points), LS&ICP (green points), and TPS&NICP (cyan points) approaches. (**a**) Human heads. (**b**) Ape crania.

The vertices of the estimated mean surfaces from every semilandmarking approach and density were submitted to separate GPA and PCA. Superimposed scatterplots of the first two PCs from each analysis are presented in Figure 10, and the proportion of the total variance explained by each axis is expressed as a percentage and tabulated in Table 3. Superimposition facilitates the visual appraisal of differences in PC scores derived using each semilandmarking approach and density.

Figure 10a shows the superimposed scatterplots of PC1 vs. PC2 from separate PCAs of the estimates of the mean surface of the human heads obtained using each semilandmarking approach. The sliding TPS and TPS&NICP approaches result in very similar PC plots, while the PCA of estimated mean surfaces generated by LS&ICP approach in a plot showing a similar pattern of variation among means, but with greater variance in both PCs (larger scatter). Similarly, for estimates of the mean surface among the ape crania derived using the sliding TPS and TPS&NICP approaches, the first two PCs from each separate PCA are superimposed in Figure 8b. These plots indicate that sliding TPS and TPS&NICP produce very similar scatters of estimated means. Both plots of Figure 10 present ‘U’-shaped curves, with the means estimated using the lowest and highest densities of semilandmarks having higher scores on PC2, although they are widely separated in PC1. 

**Figure 11:**
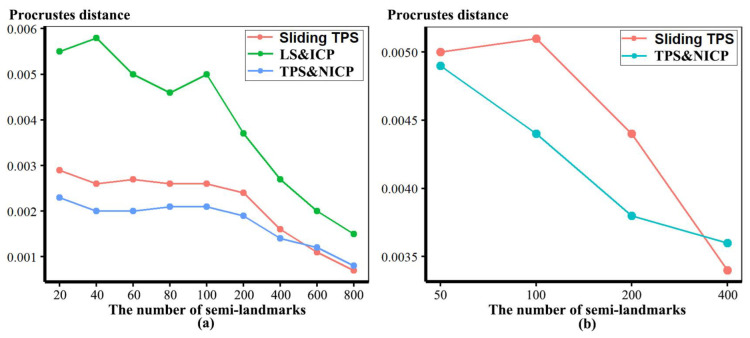
Procrustes distances of estimated mean surfaces between every density and the maximum density from each approach to semilandmarking. (**a**) Human heads. (**b**) Ape crania.

These results are supported by the Procrustes distances computed between the mean surface mesh vertices derived from each lower density and the maximum density of the semilandmarks, as shown in Table 4 and Table 5 and Figure 11. For both datasets, all semilandmarking approaches show convergence between the surfaces based on increasing numbers of semilandmarks and those based on the maximum number. For the headspace data (Table 4; Figure 11a), sliding TPS and TPS&NICP perform similarly, in that they result in mean surfaces based on <1000 semilandmarks that are closer to those based on 1000 semilandmarks than their equivalents from LS&ICP. Likewise, sliding TPS and TPS&NICP perform similarly and show convergence for the ape cranial dataset (Table 5, Figure 11b).

**Figure 12:**
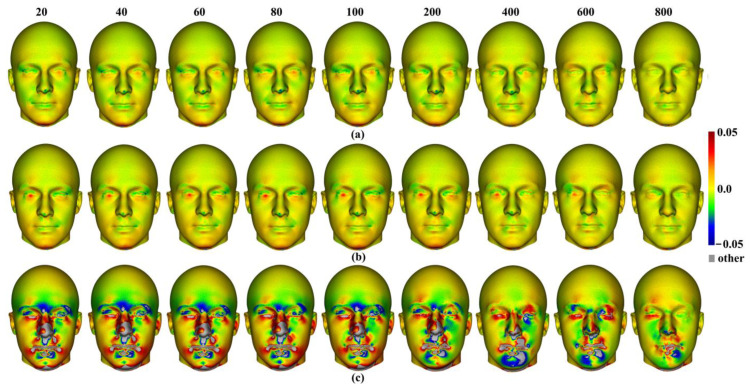
Colour maps of local surface mesh area differences between mean surfaces of human heads derived using lower densities (target) and from 1000 semilandmarks (reference) using different semilandmarking approaches. (**a**) Sliding TPS. (**b**) TPS&NICP. (**c**) LS&ICP. Scale bar indicates difference in local area between reference and target surfaces expressed as a proportion of the reference area.

Within each approach to semilandmarking, the local variations in the area between the human head mean surfaces estimated by increasing semilandmark densities and the surface from the 1000 semilandmarks were visualised as colour maps. These are presented in Figure 12a–c. Consistent with the Procrustes distances presented in Table 4, the closest fitting surfaces are between the surfaces derived using semilandmarks from sliding TPS and TPS&NICP. The colour maps comparing these surfaces with those from the 1000 semilandmarks are relatively smooth (Figure 12a,b). Further, as semilandmarking density increases, the surfaces based on lower densities of semilandmarks converge with the surface from the 1000 semilandmarks. Differences are more pronounced between surfaces derived using lower semilandmarks densities and the 1000 semilandmarks generated by the LS&ICP approach. This reflects the generally greater Procrustes distances presented in Table 4, and, visually, differences are most evident in the face (Figure 12c). The nasal, ocular, and perioral regions show localized large differences but converge with increasing semilandmarking density on the surface derived using 1000 semilandmarks, particularly around the nose and eyes. However, with increasing semilandmark densities generated by LS&ICP, the quality of the mean surfaces is poor (i.e., less sharp features around the eyes and mouth in Figure 4c) because equivalent semilandmarks lie in different anatomical locations. 

**Figure 13:**
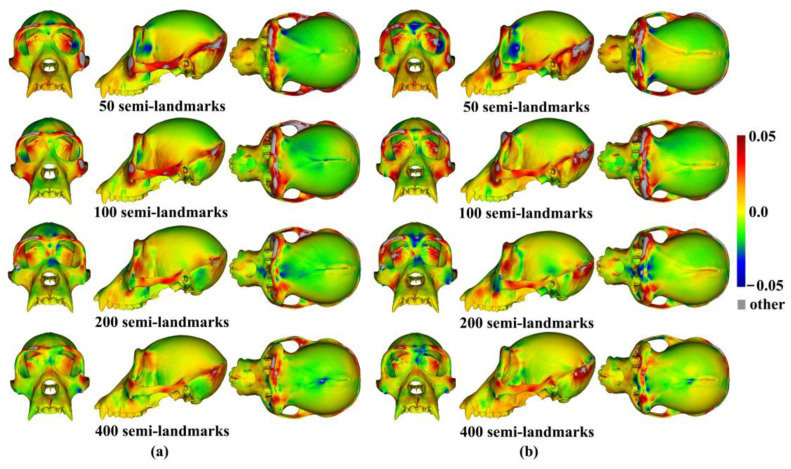
Colour maps of local surface mesh area differences among mean surfaces of ape crania derived using lower densities (target) and 800 semilandmarks (reference) based on different semilandmarking approaches. (**a**) Sliding TPS. (**b**) TPS&NICP. Scale bar indicates difference in local area between reference and target surfaces expressed as a proportion of the reference area.

Similar comparisons were undertaken for the ape crania. Figure 13a,b show regional differences in the area of mean surfaces computed between lower densities and the maximum density of 800 semilandmarks generated by the sliding TPS and TPS&NICP approaches. In both, the smallest differences are found in the cranial vault, where the colour map is smooth and indicative of small local area differences. Larger differences are observed around the frontal bone, supraorbital ridges, zygomatic arches, malar region, nasal bones, and maxillae. Consistent with Table 5, with increasing semilandmark density, a degree of convergence occurs with the surface based on the 800 semilandmarks. 

### 3.2. Comparison of Estimates of Allometrically Scaled Surfaces

We generated surface meshes warped by TPS to the predicted landmark and semilandmark configurations representing the extreme limits (maximum and minimum centroid sizes) of the allometric vector; then, after re-sliding and re-warping, we assessed the overall and regional differences between surfaces generated by different semilandmarking approaches and densities. 

#### 3.2.1. Different Semilandmarking Approaches

**Figure 14:**
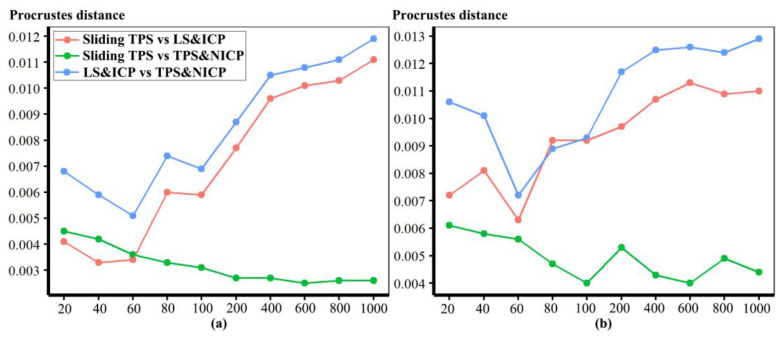
Procrustes distances computed between the vertices of human head surfaces, allometrically scaled to the maximum and minimum centroid sizes, based on different semilandmarking approaches after re-semilandmarking and re-warping the template mesh. (**a**) Maximum. (**b**) Minimum.

Procrustes distances between the vertices of the allometrically scaled surfaces of human heads representing the maximum centroid size generated by different approaches are illustrated in Figure 14a. Likewise, Procrustes distances between the fitted surfaces representing the minimum centroid size are illustrated in Figure 14b. In both cases, in comparisons between LS&ICP and the other two approaches, Procrustes distances between surface meshes increase with increasing numbers of semilandmarks, while those between sliding TPS and TPS&NICP decrease. Sliding TPS and TPS&NICP approaches result in the most similar predictions as the semilandmarking density increases. The distances between predicted shapes at minimum size are somewhat greater than those at the maximum size because of the skewed distribution of centroid sizes (see Figure 17). 

**Figure 15:**
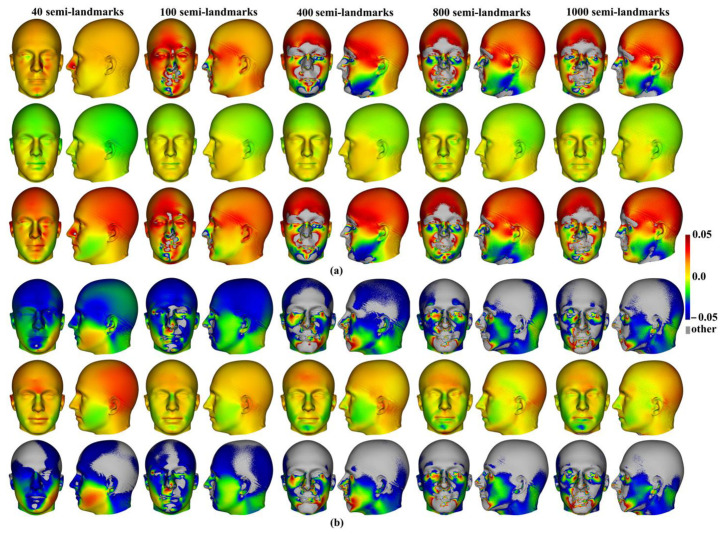
Visualization of the differences in mesh triangle surface areas between predicted allometrically scaled surfaces of human heads representing the (**a**) maximum and (**b**) minimum centroid size generated by different semilandmarking approaches. In each figure—top row: sliding TPS (reference) vs. LS&ICP (target); middle row: sliding TPS (reference) and TPS&NICP (target); bottom row: TPS&NICP (reference) vs. LS&ICP (target). Scale bar indicates difference in local area between reference and target surfaces expressed as a proportion of the reference area.

Additionally, the differences between the allometric predictions of the large and small surfaces were visualised between different semilandmarking approaches and different semilandmarks densities in Figure 15. The visualizations show differences in the surface area of the equivalent triangles among the re-warped and re-semilandmarked surface meshes predicted for the maximum centroid size in Figure 15a, and those corresponding to the minimum centroid size are illustrated in Figure 15b. In both cases, the differences between the surface mesh predictions based on landmarks and semilandmarks from sliding TPS and TPS&NICP are small (middle rows in Figure 15a,b). They reflect the Procrustes distances in Figure 14a,b in becoming more similar with an increasing semilandmark density and in being more similar for comparisons among predictions of the surface at the maximum centroid size than at the minimum. The differences between surface meshes predicted by LS&ICP and the other approaches (top and bottom rows in Figure 15a,b) also reflect the Procrustes distances in Figure 14a,b in being large, becoming larger with increasing density, and in being larger for comparisons of the predicted surfaces at the minimum centroid size. 

Similar visualisations compared allometrically scaled surfaces of the ape cranial dataset. The LS&ICP approach was not evaluated because it failed to produce sensible semilandmarks when applied to these more complex and variable surfaces. Procrustes distances between the mesh vertices of the predicted cranial surface corresponding to the maximum and minimum centroid size estimated using the sliding TPS and TPS&NICP approaches are compared in Table 6. These distances indicate that differences between both the allometric predictions of the surface increase with an increasing semilandmark density, as with the comparison of the means from the ape data estimated using the sliding TPS and TPS&NICP approaches (Table 1). In Table 6, Procrustes distances at the maximum centroid size are less than those at the minimum, consistent with the skewing of the distribution of centroid sizes towards the maximum, which results in greater allometric warping of the mean shape towards the minimum than the maximum centroid size (see Figure 18). Further, the Procrustes distances are somewhat larger than those between the estimated mean surfaces in Table 1, indicating greater differences between the allometrically scaled surfaces. 

**Figure 16:**
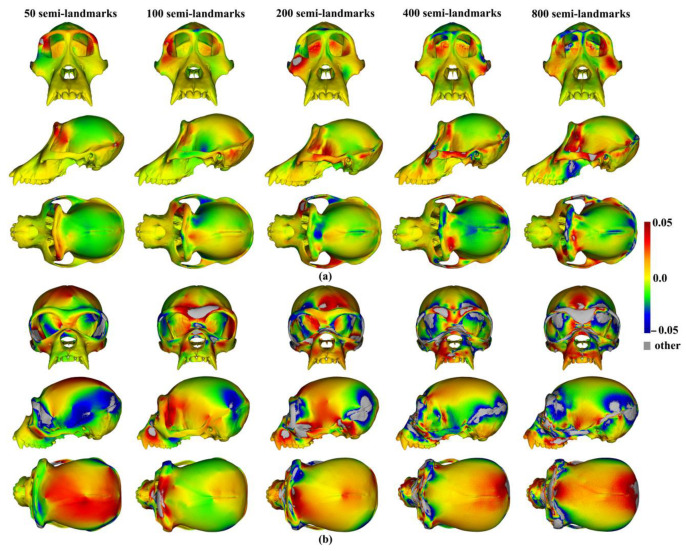
Visualization of the differences in mesh triangle surface areas between the fitted surface shapes of ape crania generated by the sliding TPS (reference) and TPS&NICP (target) approaches after re-semilandmarking and re-warping the template. (**a**) Comparison of predictions corresponding to the maximum centroid size. (**b**) Comparison of predictions corresponding to the minimum centroid size. Scale bar indicates difference in local area between reference and target surfaces expressed as a proportion of the reference area.

These differences are visualized in Figure 16 and are consistent with the Procrustes distances of Table 6; differences in mesh triangle surface areas increase with semilandmarking density, are greater for estimates of the mean ape cranium scaled to the minimum centroid size, and are more pronounced around more complex surface regions, e.g., the periorbital region, crests, and the infratemporal region. 

**Figure 17:**
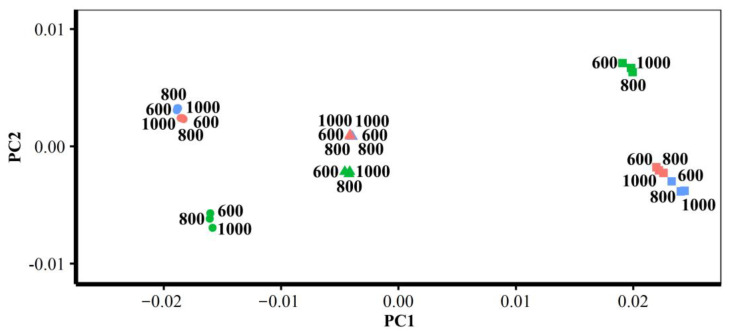
PC1 (92.4% of total variance) vs. PC2 (4.7%) from PCA of the mean and allometrically scaled head surfaces derived using varying densities of semilandmarks and each semilandmarking approach. Triangles = means, squares = allometric predictions of surfaces at the sample minimum centroid size, circles = allometric predictions of surfaces at the sample maximum centroid size. Red = sliding TPS, blue = TPS&NICP, green = LS&ICP. Numbers indicate number of semilandmarks. The sliding TPS and TPS&NICP means are nearly superimposed.

**Figure 18:**
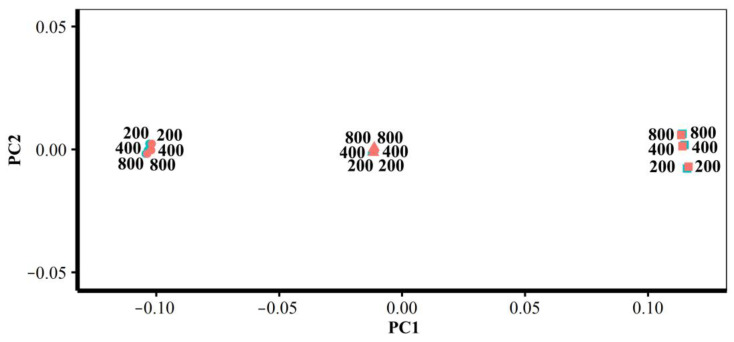
PC1 (99.5% of total variance) vs. PC2 (0.15%) from PCA of the mean and allometrically scaled head surfaces derived using varying densities of semilandmarks and the sliding TPS and TPS&NICP semilandmarking approaches. Triangles = means, squares = allometric predictions of surfaces at the sample minimum centroid size, circles = allometric predictions of surfaces at the sample maximum centroid size. Red = sliding TPS, blue = TPS&NICP. Numbers indicate number of semilandmarks. Means are nearly superimposed.

Finally, these predictions are compared through GPA and PCA of allometrically scaled mesh vertices created using varying numbers of semilandmarks from each semilandmarking approach. The first two PCs from PCAs of the mean and allometrically scaled head surfaces are presented in Figure 17, and those of the ape surfaces are in Figure 18. The first two PCs in both of these analyses account for nearly all of the variance among the surfaces (heads 97%; ape crania >99%), so they represent the differences between them well. 

Consistent with the visualisations in Figure 15 and the Procrustes distances in Table 4 and Figure 14, the PC plot of head data (Figure 17) shows that sliding TPS and TPS&NICP achieve very similar results (surfaces) with the means plotting on top of each other; the allometric predictions of the mean surface at the sample maximum centroid size (PC1 left, circles) grouping closely and those at the sample minimum centroid size (PC1 right, rectangles) being more variable. The mean and allometrically scaled surfaces from LS&ICP (green) are somewhat dissimilar based on the PC plots, Procrustes distances, and colour maps. Likewise, the PC plot of ape cranial surfaces (Figure 18) is consistent with the visualisations in Figure 16 and the Procrustes distances in Table 5 and Table 6. This shows that the mean and allometrically scaled surfaces of ape crania derived using sliding TPS and TPS&NICP are very similar to each other, with smaller variance among the predictions of surface mesh shape at the sample maximum centroid size (PC1 left, circles) than those at the sample minimum centroid size (PC1 right, rectangles). 

Further, the plots of Figure 17 and Figure 18 serve to provide perspectives on the differences seen in Table 6 and Figure 14, Figure 15 and Figure 16. While the colour maps are highly sensitive to differences in surfaces and identify many regions of difference, when they are set against the differences between the estimates of the means and allometrically scaled means in the PC plots, they appear much more similar, especially for comparisons of the results obtained using the sliding TPS and TPS&NICP approaches at all semilandmarking densities. 

#### 3.2.2. Different Densities of Semilandmarks

**Figure 19:**
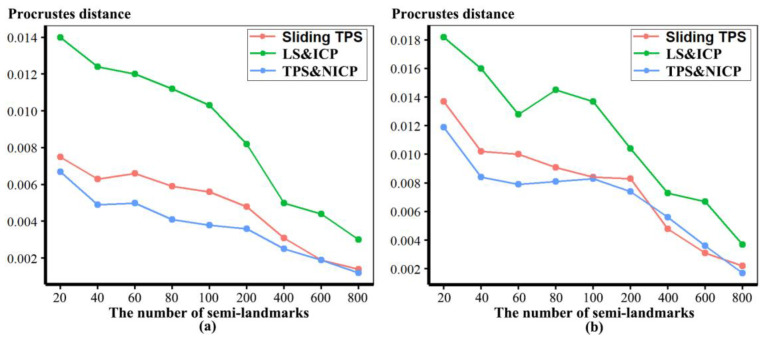
Procrustes distances, after re-semilandmarking and re-warping, between the allometrically scaled head surfaces derived from the maximum density and those from lower semilandmark densities. (**a**) Procrustes distances between predicted surfaces at the maximum centroid size. (**b**) Procrustes distances between predicted surfaces at the minimum centroid size.

For each semilandmarking approach and dataset, the differences in shape between the allometrically scaled surfaces derived from lower semilandmark densities and those from the maximum density were assessed by computing the Procrustes distances between their vertices and visualizing differences in local surface areas. For the head surfaces, Table 7 presents and Figure 19 plots these Procrustes distances. In both cases, the sliding TPS and TPS&NICP approaches consistently result in surfaces from lower semilandmarking densities being more similar (smaller Procrustes distances) to the surface with the maximum semilandmarking density than for those derived using the LS&ICP approach. Further, at lower semilandmarking densities, distances from the TPS&NICP approach are slightly smaller than those from sliding TPS. Procrustes distances are a little larger among the predicted surfaces at the sample minimum centroid size, especially at lower semilandmarking densities, than among those at the sample maximum centroid size because of the skewed distribution of centroid sizes (see Figure 17). 

**Figure 20:**
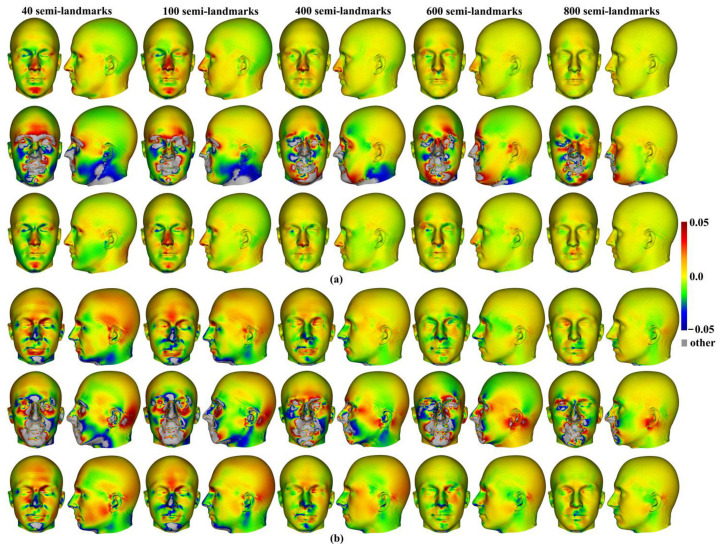
Colour map of local area differences computed between the re-semilandmarked and re-warped allometric predictions of surfaces of human heads at the (**a**) maximum and (**b**) minimum sample centroid sizes, computed between lower densities (reference) and the maximum density (target) of semilandmarking. In each figure—top row: sliding TPS; middle row: LS&ICP; bottom row: TPS&NICP. Scale bar indicates difference in local area between reference and target surfaces expressed as a proportion of the reference area.

Additionally, local differences in the area between the allometric predictions of head surfaces derived using the maximum density semilandmarks and lower densities from each semilandmarking approach are visualized in the colour maps in Figure 20. Figure 20a,b present the visualisations corresponding to the sample maximum and minimum centroid size, respectively. Consistent with the Procrustes distances presented in Table 7 and Figure 19, the LS&ICP approach shows the greatest differences between surfaces derived from lower densities and the maximum, while sliding TPS and TPS&NICP perform similarly. In all cases, shape differences between lower and maximum semilandmarking densities become smaller with increasing density. The greatest differences between semilandmarking densities are found around the nose, mouth, ears, and chin, where the topography is complex, and the smallest are found around the forehead and scalp, where the surface is smooth and lacks identifiable landmarks. 

**Figure 21:**
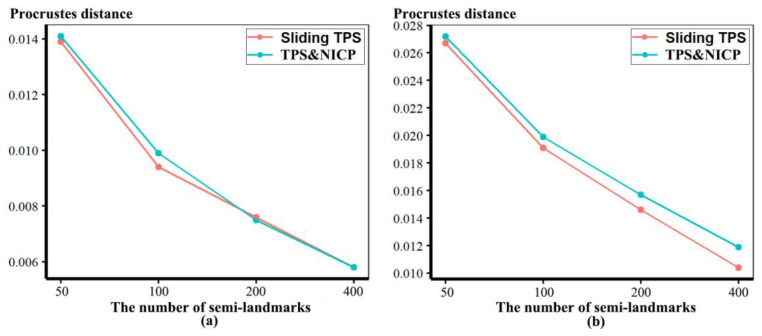
Procrustes distances, after re-semilandmarking and re-warping, between the allometrically scaled ape cranial surfaces derived from the maximum density and those from lower densities of semilandmarks. (**a**) Procrustes distances between predicted surfaces at the maximum centroid size. (**b**) Procrustes distances between predicted surfaces at the minimum centroid size.

These analyses were repeated using the allometric predictions of ape cranial surfaces between every density and the maximum density of semilandmarks generated by sliding TPS and TPS&NICP, respectively. The Procrustes distances between the allometrically scaled predictions of the ape crania from varying semilandmarking densities and those from the maximum semilandmarking density are presented in Table 8 and plotted in Figure 21. These are very similar in magnitude for surfaces derived using both sliding TPS and TPS&NICP approaches at all densities and, with increasing density, show a similar trend of convergence on the surface derived using 800 semilandmarks. Procrustes distances between this surface and those derived using lower density semilandmarks are greater for estimates of the allometric predictions of surfaces at the minimum centroid size than at the maximum. This reflects the skewed distribution of centroid sizes, in particular, the greater difference between the overall mean and the predicted mean surface at the minimum than at the maximum centroid size (see Figure 18). 

**Figure 22:**
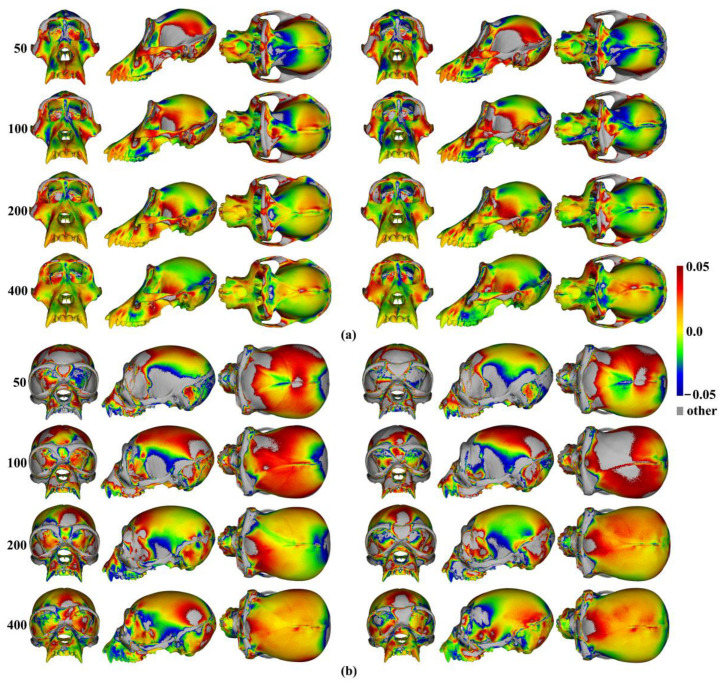
Colour map of local area differences computed between the re-semilandmarked and re-warped allometric predictions of surfaces of ape crania at the (**a**) maximum and (**b**) minimum sample centroid sizes, computed between lower densities (reference) and the maximum density (target) of semilandmarking. Left: sliding TPS; right: TPS&NICP. Scale bar indicates difference in local area between reference and target surfaces expressed as a proportion of the reference area.

These localised variations in the surface areas of the allometrically scaled surfaces are visualised in Figure 22. These visualisations reflect the Procrustes distances presented in Table 8 and Figure 21 in showing greater differences between semilandmarking densities for the allometric predictions of the ape crania at the minimum centroid size than those at the maximum and convergence with an increasing semilandmarking density. The largest shape differences are observed in the facial region; the zygomatic arches; and the supraorbital, temporal, and nuchal regions, where surface topography is most complex, and the least are observed over the cranial vault. 

### 3.3. Comparisons of Mean and Allometrically Scaled Surface Resulting from Landmarks Alone

**Figure 23:**
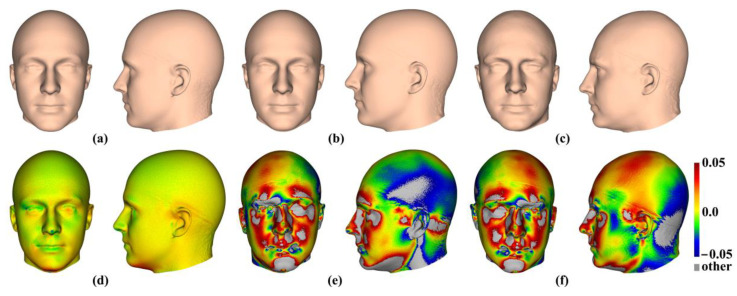
(**a**) Mean head surface estimated by warping the template surface to the mean configuration of landmarks and 1000 semilandmarks from sliding TPS. (**b**) Mean head surface estimated by warping the template surface to the mean landmark configuration. (**c**) Mean head surface estimated by warping the surface of the head with minimum Procrustes distance from the mean to the mean landmark configuration. (**d**) Colour map between surfaces a (reference) and b (target). (**e**) Colour map between surfaces a (reference) and c (target). (**f**) Colour map between surfaces b (reference) and c (target).

**Figure 24:**
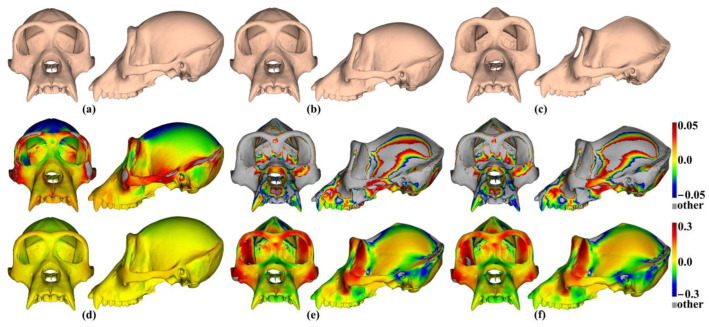
Top row: (**a**) Mean ape cranial surface estimated by warping the template surface to the mean configuration of landmarks and 1000 semilandmarks from sliding TPS. (**b**) Mean ape cranial surface estimated by warping the template surface to the mean landmark configuration. (**c**) Mean ape cranial surface estimated by warping the surface of the cranium used to generate the template to the mean landmark configuration. Colour maps between the surfaces using different colour ranges (see text) in the middle row and bottom rows: (**d**) Colour map between surfaces a (reference) and b (target). (**e**) Colour map between surfaces a (reference) and c (target). (**f**) Colour maps between surfaces b (reference) and c (target).

For each dataset, the mean surface from the sliding TPS and 1000 semilandmarks was compared with warped surfaces derived using only the landmarks. The template surfaces, which are themselves an initial estimate of the average surface (see Methods), were fitted to the mean landmarks (Figure 23 for heads and Figure 24 for apes). In practice, it is common to use the surface of an individual close to the mean for visualisation, yet the effects of the choice of surface are unclear. Therefore, the estimation of mean surfaces was repeated, this time using the head surface with the smallest Procrustes distance to the mean and the ape surface used to generate the ape template. The resulting predictions of mean head surfaces for each dataset were compared using colour maps of local mesh surface area changes (Figure 23). For the ape surface comparison, two different colour maps were drawn, the first using the same colour scale range used in the preceding analyses to allow for a direct comparison with them and the second using an extended range to better visualise the full range of local area differences (Figure 24). 

To the naked eye, the mean head surfaces (Figure 23a–c) differ, but to a lesser degree than the mean ape cranial surfaces (Figure 24a–c). In both cases, the greatest similarity (d, in each figure) is between (a), the surface estimated by warping the template surface to the mean configuration of landmarks and the 1000 semilandmarks from sliding TPS, and (b), the surface estimated by warping the template surface to the mean landmark configuration. More marked differences (e) are found by comparing (a) with (c). In the case of the head dataset, both comparisons with Figure 23c show very similar differences in the face (Figure 23e compared with Figure 23f), especially around the nose, mouth, and eyes where landmarks are present, but there are contrasting differences over the scalp, which lacks identifiable landmarks to control warping. In the case of the ape cranial dataset, where landmarks are distributed over the entire surface, both comparisons with Figure 24d are similar (Figure 24e compared with Figure 24f), with the main differences concentrated around crests and ridges.

**Figure 25:**
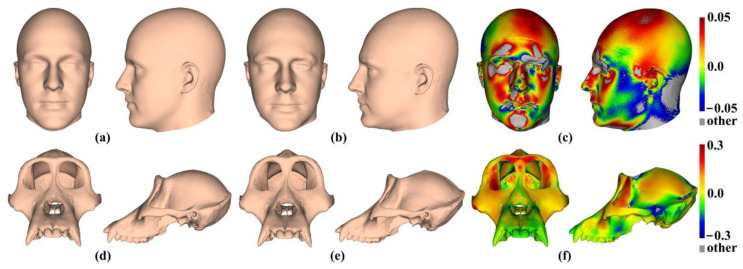
(**a**) Allometric prediction of head surface at the sample maximum centroid size using the template surface, estimated using landmarks and 1000 semilandmarks from sliding TPS. (**b**) Allometric prediction of head surface at the sample maximum centroid size using the surface of the head with minimum Procrustes distance to the mean warped using landmarks alone. (**c**) Colour map between surfaces a (reference) and b (target). (**d**) Allometric prediction of ape surface at the sample maximum centroid size using the template surface, estimated using landmarks and 800 semilandmarks from sliding TPS. (**e**) Allometric prediction of ape surface at the sample maximum centroid size using the ape cranium used to generate the template, estimated using landmarks alone. (**f**) Colour maps between surfaces d (reference) and e (target) using different ranges.

**Figure 26:**
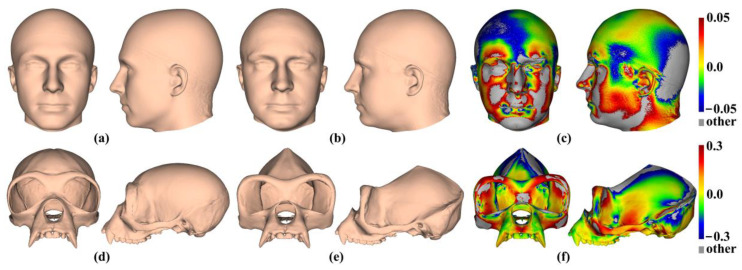
(**a**) Allometric prediction of mean head surface at the sample minimum centroid size using the template surface, estimated using landmarks and 1000 semilandmarks from sliding TPS. (**b**) Allometric prediction of mean head surface at the sample minimum centroid size using the surface of the head with minimum Procrustes distance to the mean warped using landmarks alone. (**c**) Colour map between surfaces a (reference) and b (target). (**d**) Allometric prediction of mean ape surface at the sample minimum centroid size using the template surface, estimated using landmarks and 800 semilandmarks from sliding TPS. (**e**) Allometric prediction of mean ape surface at the sample minimum centroid size using the ape cranium used to generate the template, estimated using landmarks alone. (**f**) Colour maps between surfaces d (reference) and e (target) using different ranges.

Similar analyses are conducted to assess how landmarks alone perform in predicting allometrically scaled surfaces, just as they might be carried out where no initial estimate of the mean surface is possible (e.g., hand-collected landmark data), but a surface mesh is available for warping. These focus on the comparison of the surface of the individual head closest to the mean and the ape surface used to generate the template, warped to the allometrically scaled landmark configurations, with those from the allometric scaling of the template surfaces based on all landmarks and the maximum densities of the semilandmarks. The results for the predictions of surfaces at the maximum sample centroid sizes are presented in Figure 25, and the minimum centroid sizes are in Figure 26. Note that the scale bar used to compare ape cranial means is wider than those used elsewhere because the differences are greater. In both cases, the surfaces of individuals warped to fit the allometrically scaled landmark configurations (Figure 25b,e and Figure 26b,e), are superficially similar to those derived by warping the template to the allometrically scaled landmark and the highest density semilandmark configurations for each dataset. However, they differ in detail such that the human head surfaces estimated using landmarks alone and the surface of the individual nearest to the mean (Figure 25b and Figure 26b) present more rounded faces with subtle differences around the eyes, mouth, and nose when compared with the template surfaces warped to the allometrically scaled landmark and semilandmark configurations (Figure 25a and Figure 26a). The same comparisons for the ape crania (Figure 25d vs. Figure 25e and Figure 26d vs. Figure 26e) present more obvious differences, particularly around sagittal and nuchal crests, orbits, and temporal fossae. 

**Figure 27:**
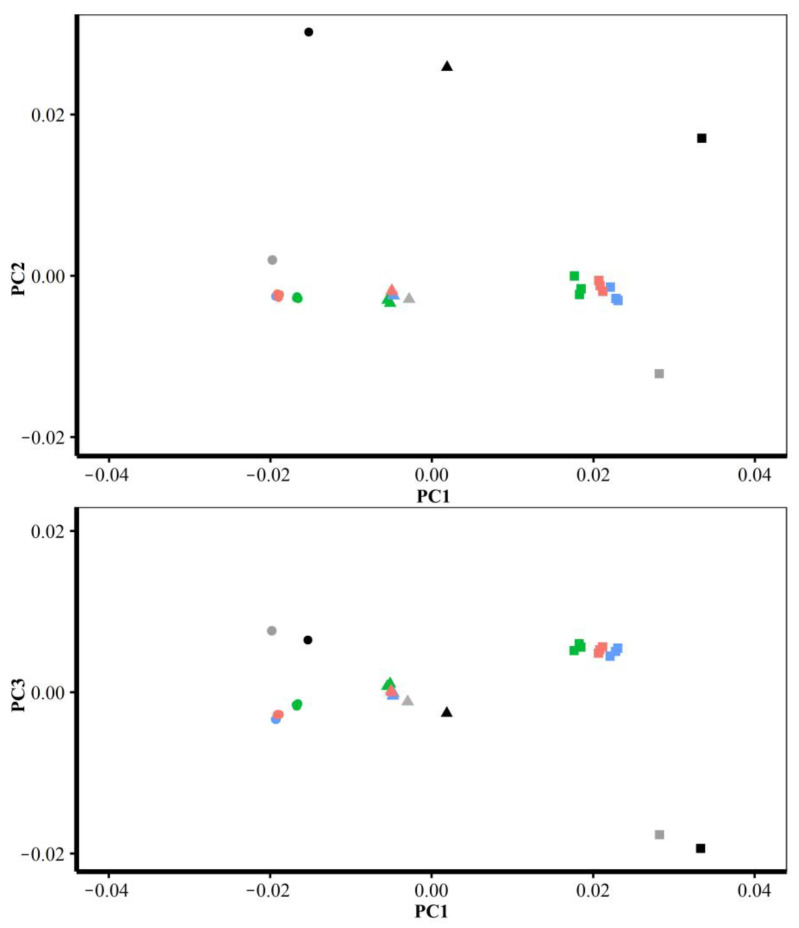
Top: PC1 (72.1% of total variance) vs. PC2 (17.5%); bottom: PC1 vs. PC3 (5.36%) from PCA of the mean and allometrically scaled head surfaces derived using varying semilandmark densities from each semilandmarking approach (from Figure 17). Red = sliding TPS, blue = TPS&NICP, green = LS&ICP. Also included in this PCA are surfaces warped to the mean and scaled landmark configurations; the head surface with minimum Procrustes distance from the mean (black); and the template surface (grey). Triangles = means, squares = allometric predictions of surfaces at the sample minimum centroid size, circles = allometric predictions of surfaces at the sample maximum centroid size.

These warped surfaces were added to the PCAs of the mean and allometrically scaled head surfaces derived using varying densities of semilandmarks and each semilandmarking approach in Figure 17 and Figure 18. Figure 27 presents plots of PC1 vs. PC2 and PC1 vs. PC3 (accounting for 95% of the total variance) of the mean and allometrically scaled head surfaces. A further 3% of the total variance is explained by PC4. The plot of PC1 vs. PC4 from this analysis (not shown) is very similar to that of Figure 17, indicating a difference in the allometric vector direction between LS&ICP and all other approaches. It is clear that the surfaces estimated by warping the surface of the individual head with the minimum Procrustes distance from the mean to the mean and allometrically scaled landmark configurations are distinct from those estimated using the template surface and semilandmarks. Additionally, the vector connecting this mean and the allometrically scaled means is not parallel to the vector connecting the semilandmark-derived mean and scaled surfaces. Further, the template surfaces warped to fit the mean and scaled landmark configurations are arranged along a vector parallel to them, but with the mean near the mean of the surfaces warped using semilandmarks. Thus, while these surfaces are warped to exactly fit the overall mean and the allometrically scaled mean landmark configurations, the regions between the landmarks are deformed in the same way for both surfaces but differently to the template surface warped to fit the landmark and semilandmark configurations. This is consistent with the visual comparisons in Figure 23a vs. Figure 23c, Figure 25a vs. Figure 25b, and Figure 26a vs. Figure 26b. 

**Figure 28:**
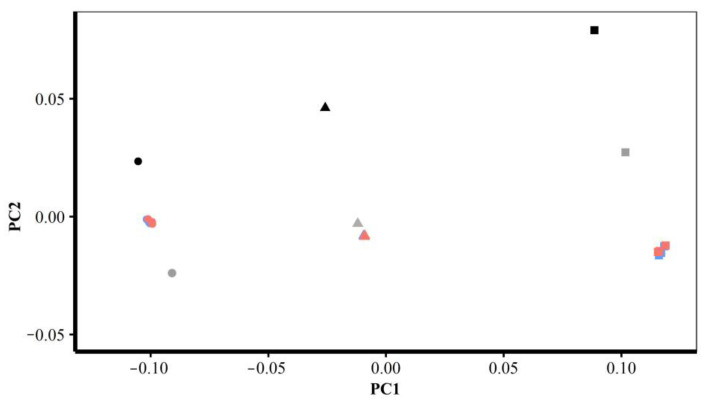
PC1 (93.2% of total variance) vs. PC2 (6.04%) from PCA of the mean and allometrically scaled ape cranial surfaces derived using varying semilandmark densities and each semilandmarking approach (from Figure 18). Red = sliding TPS; blue = TPS&NICP. Also included in this PCA are surfaces warped to the mean and scaled landmark configurations; the ape cranial surface used to generate the template (black); and the template surface (grey). Triangles = means, squares = allometric predictions of surfaces at the sample minimum centroid size, circles = allometric predictions of surfaces at the sample maximum centroid size.

Similarly, Figure 28 presents a plot of PC1 vs. PC2 of the mean and allometrically scaled ape cranial surfaces. This plot accounts for 99% of the total variance. The surfaces estimated by warping the ape cranial surface used to generate the template and allometrically scaled landmark configurations are again distinct from those using the template surface and semilandmarks. As with the head surfaces, the vector connecting the semilandmark-derived mean and allometrically scaled surfaces is not parallel to the vector connecting these estimates of the mean and allometrically scaled surfaces, and the surfaces obtained by warping the template to fit the mean and allometrically scaled landmark configurations lie along a parallel vector to the latter, with the mean near the means of surfaces derived using semilandmarks. Thus, as with the head surfaces, the surface between the landmarks is different to the template surface warped to fit the landmark and semilandmark configurations, and it is deformed differently. Again, this is consistent with the visual comparisons of Figure 24a vs. Figure 24c, Figure 25d vs. Figure 25e, and Figure 26d vs. Figure 26e. 

## 4. Discussion

The use of digital surface meshes of biological and anthropological specimens in 3D GM studies has become increasingly common, as has the use of landmarks and semilandmarks generated by different semilandmarking approaches in order to compare the details of morphology [10,13,18,21,34,40]. While dense coverage by semilandmarks allows for more detailed descriptions of form and, potentially, biological signals [41], it introduces several difficulties in comparing forms. Further, given that semilandmarks are treated as equivalent between specimens in GM analyses and are given the same weight as landmarks, the basis of equivalence is an important consideration. In studies of biological transformations such as those that occur during development and evolution, the equivalences required to model and compare them are developmental or evolutionary. Landmarks and semilandmarks at each stage need mark-up points that are equivalent between specimens in terms of development or evolution at another stage (homologous points). For landmarks, this matching is based on prior knowledge, but for semilandmarks, it is algorithmic and relies on mathematical mappings and topographical features. As such, the extent to which semilandmarks can be considered homologous has contributed to the debate about their validity and usefulness in relation to the study of developmental or evolutionary transformations [6,7,41]. 

It has been noted by previous researchers that because the locations of semilandmarks on surfaces and curves are uncertain they should not be interpreted singly, but rather as a whole [3,6,29]. While this avoids overinterpreting differences in individual semilandmark locations, it does not avoid statistical issues. Thus, differences in semilandmark locations will lead to different distance matrices among specimens and, thus, to different analytical results. The extent of this issue has been explored in several previous studies [11,13,18,19,20,21,22]. Additionally, the use of high-density semilandmarks raises statistical issues related to the ratio of variables to specimens (i.e., high *p* and low *n*) and in assessing covariances within landmark and semilandmark configurations [7,42]. 

Statistical considerations aside, high-density semilandmarks are routinely used to assess shape variations and covariations and to perform classification [41,43,44,45], with results presented as visualisations of a warped surface mesh. It is, therefore, of interest to know how different semilandmarking approaches and semilandmark densities affect visualisations. This has been addressed by the analyses presented here. 

In this study, we compare surface meshes warped to configurations of landmarks and semilandmarks arising from GM analyses that represent the overall mean and allometrically scaled surfaces. The aim is to compare the surface meshes used for visualisation rather than the statistical outcomes of analyses of the landmark and semilandmark configurations. These were compared in [24]. Three different semilandmarking approaches were used with varying semilandmark densities. These are the method of sliding semilandmarks, minimising the bending energy of a set of thin-plate splines or Procrustes distances [37], the non-rigid combined approach of TPS&NICP [26], and the rigid LS&ICP approach. These lead to semilandmark configurations that differ in the locations of semilandmarks. These differences are smallest between sliding the TPS and TPS&NICP approaches and larger when comparing these with the LS&ICP approach (Figure 2). However, the locations of individual semilandmarks are not interpretable and, as noted above, they lie on the surface and so should be interpreted as a whole in terms of the differences between surfaces that fit them. 

This study aimed to achieve this by empirically testing two hypotheses using surface scans of human heads and ape crania: that there are no differences in surface mesh shape between estimates derived using different semilandmarking densities and approaches applied to surfaces representing (a) the mean of a sample and (b) allometrically scaled shapes. The surfaces were quantitatively compared using the coordinates of their vertices after re-semilandmarking and re-warping (see Methods) to calculate Procrustes distances between them and, where relevant, by extracting and comparing principal components. They were visually compared using colour maps of differences in local surface areas. Both hypotheses are falsified; differences clearly exist between estimated mean and allometrically scaled surfaces, but the degree of difference between semilandmarking approaches is small to moderate, with the non-rigid semilandmarking approaches (sliding TPS and TPS&NICP) showing a high degree of consistency. 

Because landmarks have more secure homology than semilandmarks and should be chosen with respect to the question at hand [6,7], they are likely few in number and less likely to result in statistical issues arising from large numbers of variables relative to the number of specimens. Additionally, surfaces can be warped to landmarks to visualise analytical results, albeit with less detail than warping based on dense correspondences. Thus, the present study also assessed differences between warped surfaces based on landmarks and semilandmarks and those based on the landmark configuration alone, using different reference surfaces. 

Three semilandmarking approaches were used to estimate the sample mean surface meshes by warping the template (an initial estimate of the average surface in each dataset) to the mean landmark and semilandmark coordinates arising from each method using varying semilandmark densities. For the head surfaces, the means are visually quite similar (Figure 4) but differ in detail (Figure 6 and Figure 7). The resulting mean surfaces from sliding TPS and TPS&NICP are the most similar, and those from LS&ICP are the most different. Similar results are obtained in estimating the mean surface of the ape crania (Table 1 and Figure 8 and Figure 9), but the LS&ICP approach performed poorly in locating semilandmarks in reasonably corresponding locations with the more complex ape cranial surfaces. In both datasets, estimated mean surfaces converge with increasing semilandmarking density on the surface from the highest density (Table 4 and Table 5 and Figure 10, Figure 11 and Figure 12). For the head surface data, warping the template surface to the mean landmark configuration (Figure 23b) resulted in a surface that was quite similar in general to that warped to landmarks and high-density semilandmarks, but differed in detail from the semilandmark-based mean (Figure 23d). This similarity is in large part due to the fact that the template surface is already an initial estimate of the mean. Repeating the analysis using the surface of the individual nearest to the mean landmarks and semilandmarks resulted in an estimate of the mean surface (Figure 23c) that presented greater differences from the semilandmark-based mean surface (Figure 23e). Visually, this approach worked reasonably despite the lack of identifiable landmarks to guide the warping of the scalp; however, this is likely because the template scalp was not an initial estimate of, and very similar to, the mean. 

The mean ape surfaces estimated using sliding TPS and TPS&NICP with varying densities of semilandmarks are also visually quite similar (Figure 8), although the surface from LS&ICP shows some obvious differences. Focusing on sliding TPS and TPS&NICP, the mean surfaces resulting from these methods using varying numbers of semilandmarks are very similar, with differences increasing with semilandmarking density, especially where surface topography is complex (Figure 9 and Table 1). Surfaces estimated using increasing numbers of semilandmarks converge on the surface estimated using the maximum number of semilandmarks (Figure 10, Figure 11 and Figure 13). 

It should be noted that in the implementation of NICP used here, the initial registration of surfaces between the template and target uses a triplet of TPS. This is also the case for the sliding TPS approach. This shared initial, non-rigid registration doubtless contributes to the similarities in results obtained using these approaches when compared to the rigid, least-squares registration employed in the LS&ICP approach. However, even the LS&ICP approach used the same landmark set for registration. It would be of interest in future work to assess the impact of using different landmark configurations to estimate semilandmarks. 

Using the mean landmark configuration alone to warp the template surface mesh results in a visually similar surface to the mean based on landmarks and high-density semilandmarks, but it differs in detail, especially around crests and ridges (Figure 24a,b,d). Visualising the mean by warping the ape surface used to generate the template results in a more different surface (Figure 24c,e,f), which, in some ways resembles the mean based on landmarks and high-density semilandmarks (Figure 24a), but it differs particularly in regions with complex topography (Figure 24c,f). These landmark-based warpings differ in detail from the landmark and semilandmark-based ones, but they also bear a resemblance. Whether or not they are adequate depends on the purpose to which they are put. They may be sufficient to describe general aspects of shape variation but would likely yield different results if used to build finite element models (FEM). The warping of a surface that is an initial estimate of the mean to the landmarks alone inevitably yields a surface more like that based on landmarks and semilandmarks than warping a surface from an individual, even if close to the mean. This also applies to comparisons of mean surfaces resulting from semilandmarking approaches and densities.

The predicted allometrically scaled mean surfaces were also compared among semilandmarking approaches and densities. With the head surface dataset, sliding TPS and TPS&NICP produced very similar surfaces, particularly at the highest semilandmarking densities (Figure 14 and Figure 15). The surfaces from LS&ICP were dissimilar. Likewise, for ape cranial surfaces, the allometrically scaled mean surfaces from sliding TPS and TPS&NICP are similar but differ in detail, especially around ridges and crests (Table 6 and Figure 16). They become more dissimilar in the regions of crests and ridges as semilandmarking density increases, reflecting the more detailed control of warping from greater semilandmark densities. Both semilandmarking approaches show a similar pattern of convergence on the surface derived from the highest density, of surfaces with increasing densities of semilandmarking (Figure 21 and Figure 22, and Table 8). 

These differences among allometrically scaled means from both datasets and the different approaches and densities of semilandmarking are summarized by the PC plots in Figure 17 and Figure 18. Figure 17 presents for the head surface data, the first two PCs from an analysis of the mean, and allometrically scaled mean surfaces derived from varying densities of semilandmarks and each approach. It shows that sliding TPS and TPS&NICP achieve very similar results, with many points overlapping, but LS&ICP results in quite different estimates of the same surfaces, which vary along a different vector from the other two approaches. The comparable analysis for the ape crania compared only sliding TPS and TPS&NICP, and the resulting PC plot shows that these achieve very similar results. These findings provide a perspective on the differences identified in the Procrustes distance matrices and visual comparisons in the analyses described above. Thus, the Procrustes distances between the mean surfaces from varying the semilandmarking approaches and densities are small compared with those between surfaces allometrically scaled to the maximum and minimum sample centroid sizes. The colour maps are very sensitive, identifying and emphasising what are, in reality, very small differences. 

Allometrically scaled ape cranial surfaces from sliding TPS with 800 semilandmarks are compared with surfaces derived by warping the template surface and the surface used to generate the template to the allometrically scaled landmark configurations. The resulting predictions of surfaces at both the sample maximum and minimum centroid sizes share general similarities with, but differ in detail from, the surfaces based on semilandmarks (Figure 25d,e,f and Figure 26d,e,f). As with the head surfaces, these differences reflect similar aspects of scaling, which may be adequate in describing general scaling trends but would likely lead to differences in FEA results among models based upon them.

The differences in scaling are emphasised by the PCAs of Figure 27 and Figure 28, where, for both datasets, the surfaces derived by warping the surface of the individual nearest to the mean to the allometrically scaled mean landmark configurations are distant from the semilandmark-based surfaces and are arranged along a vector that is not parallel to the vector between surfaces scaled using semilandmarks. Warping the template surface to the mean and allometrically scaled means in both datasets results in a vector parallel to that derived by warping the head surface of the individual nearest to the mean or the ape cranium used to generate the template, but with the mean close to the means from the semilandmark-based approaches. This indicates that these different surfaces scale in very similar ways. Thus, the choice of template surface determines where in the shape space the allometric vector is located while the landmarks and semilandmarks used to deform the surface determine how it is deformed. Semilandmarks result in the surface regions between landmarks being deformed differently from what is achieved by warping to the landmark configurations alone. This is not surprising and underlines how semilandmarks contribute to controlling surface deformations. 

The results of this study show that different semilandmarking approaches and densities achieve different visualisations of mean and allometrically scaled surfaces. The degree of difference depends on the approach, with non-rigid semilandmarking (sliding TPS and TPS&NICP) producing surfaces that are consistently more similar to each other than to those derived using the rigid LS&ICP approach. Additionally, the non-rigid approaches show consistency in the surfaces produced using semilandmarks of varying densities. While Procrustes distances and colour maps emphasise differences among the approaches, PCAs comparing the scaled mean surfaces show that the differences between surfaces from non-rigid semilandmarking approaches are very small when compared to the differences among allometrically scaled means. The differences between surfaces derived using LS&ICP are greater. 

Semilandmarking involves a great deal of extra effort compared with landmarking alone, and, as has been noted earlier, brings with it some severe statistical issues. This has led to the questioning of their benefits and criticism stating that they may lead to erroneous conclusions [7,42]. Thus, this study compared surfaces warped using landmark configurations alone with those from landmark and semilandmarking configurations. These comparisons have shown that if a surface that is an initial estimate of the mean surface is used then the mean surfaces are well estimated. This is to be expected since the mean landmarks have little warping to do. This finding likely explains why LS&ICP results in more similar mean surfaces to those from sliding TPS and TPS&NICP at lower rather than higher semilandmarking densities (Figure 6 and Figure 7). When an alternative surface is used, the surface visualisation is different, having inherited features from this new surface. Surfaces warped to scaled landmark configurations show differences and some similarities to those warped to landmarks and semilandmarks in combination. Such analyses and visualisations based on landmarks alone may be perfectly adequate for many questions; they involve less work to produce and avoid the statistical issues that can arise with many semilandmarks and few specimens. However, compared with surfaces from semilandmarks, they would likely lead to different results if used to build finite element models. 

Finally, we should emphasise that consistency is not the same as accuracy [7]. It is tempting to conclude that the remarkable consistency of surface shapes derived using sliding TPS and TPS&NICP reflects accuracy in the estimation of means. Our results cannot, however, support or refute this possibility since no ‘true mean’ is known (or knowable). Estimates of means depend on what quantities are measured and compared because means are a statistical, rather than biological, entity, particular to the data used to calculate the mean. The results are ‘correct’ for the variables (semilandmark locations) resulting from each method. However, with semilandmarks, there is inevitable uncertainty about the extent to which they are equivalent between specimens in terms of homology. Our studies show that differences in semilandmark locations among specimens will lead to differences in statistical results [24] and visualisations (present study). In these studies, these differences are quite small relative to the differences among specimens, but it is not clear to what extent these empirical results apply to diverse datasets and semilandmarking approaches (e.g., minimisation of Procrustes distances by sliding [10]; morphometric ‘fishnets’ [46]). This can only be addressed by further extensive studies of real data and through simulation experiments, in which an initial ‘mean’ is perturbed and then estimated from the perturbed data. 

For now, we have shown that the two non-rigid semilandmarking approaches yield consistent estimates of mean and scaled surfaces. Semilandmarking involves a great deal of additional work and runs statistical risks in analyses. With these things in mind, the investigator should carefully consider if semilandmarking is necessary to answer the question at hand and balance this need against the statistical and biological (e.g., regarding homology) downsides and the time involved in gathering and using semilandmarks to assess shape variances and covariances. It may be a more secure strategy to base statistical tests on homologous landmarks and visualisations on landmarks and semilandmarks from parallel analyses. 

It should be borne in mind that homology is often also uncertain for landmarks and that different sets of landmarks will lead to different results. However, the three approaches that we compared in this study led to visually similar estimates of surface meshes that may be adequate for visualisation and functional simulation, in the sense that they are likely to be fair representations of average and scaled surfaces, but there is no single ‘true’ representation against which to assess this (see above). Their applicability depends on how much error in the estimation of the surface shapes is judged acceptable given the context of the particular study. 

Finally, it should be noted that this study is limited in its scope; being based on only human heads and ape crania, different datasets need to be examined to assess the reliability of the findings. Studies also need to be conducted using simulated data in which true mean and allometrically scaled surfaces are known in order to assess the accuracy of the estimates of these surfaces. Additionally, this study compared a limited range of possible approaches to semilandmarking, and future work needs to extend these comparisons to include other methods and ‘landmark-free’ approaches. 

## 5. Conclusions

This study examined the effects of different semilandmarking approaches and semilandmarking densities on estimates of mean and allometrically scaled mean surfaces. These were investigated by assessing overall and regional shape differences based on Procrustes distances and colour maps of local surface mesh area differences. The results show that the mean and fitted surfaces generated by the sliding TPS and TPS&NICP approaches are very similar, while the LS&ICP approach yields surfaces that differ most. Surfaces warped to landmark configurations differ from these depending on the degree of similarity of the surface to the mean and show a different vector of allometric scaling, reflecting the differences between TPS interpolation and the semilandmark control of surfaces between landmarks. In conclusion, visualisations derived using semilandmarks from non-rigid semilandmarking approaches especially are likely to fairly represent surfaces and differences between them, but they are not identical. The extent to which these differences are important depends on the particular study context and aims. 

## Figures and Tables

**Table 1:** Procrustes distances computed between the mean surfaces of ape crania generated by sliding TPS and TPS&NICP after re-semilandmarking and re-warping the template mesh.

	50	100	200	400	800
dist	0.0018	0.0025	0.0024	0.0027	0.0030

**Table 2:** Procrustes distances computed between the mean landmarks and semilandmarks of ape crania generated by sliding TPS and TPS&NICP after re-semilandmarking the warped mesh.

	50	100	200	400	800
dist	0.0023	0.0029	0.0029	0.0032	0.0034

**Table 3:** Percentages of total variance explained by PC 1 and PC 2 of the mean surface shape.

	Human Heads		Ape Crania	
	PC1	PC2	PC1	PC2
Sliding TPS	79.96%	7.37%	42.12%	27.51%
LS&ICP	68.32%	9.68%	-	-
TPS&NICP	64.15%	13.48%	42.33%	24.76%

**Table 4:** Procrustes distances between the vertices of the estimated mean human head surfaces using 1000 semilandmarks and those using increasing numbers of semilandmarks from each approach after re-semilandmarking and re-warping the template mesh.

	20	40	60	80	100	200	400	600	800
Sliding TPS	0.0029	0.0026	0.0027	0.0026	0.0026	0.0024	0.0016	0.0011	0.0007
LS&ICP	0.0055	0.0058	0.0050	0.0046	0.0050	0.0037	0.0027	0.0020	0.0015
TPS&NICP	0.0023	0.0020	0.0020	0.0021	0.0021	0.0019	0.0014	0.0012	0.0008

**Table 5:** Procrustes distances between the vertices of mean ape cranial surfaces estimated by each approach using 800 semilandmarks and those estimated using increasing numbers of semilandmarks after re-semilandmarking and re-warping the template mesh.

	50	100	200	400
Sliding TPS	0.0050	0.0051	0.0044	0.0034
TPS&NICP	0.0049	0.0044	0.0038	0.0036

**Table 6:** Procrustes distances computed between vertices of ape cranial surfaces allometrically scaled to the maximum (Max) and minimum (Min) centroid size from sliding TPS and TPS&NICP semilandmarking approaches after re-semilandmarking and re-warping the template mesh.

	50	100	200	400	800
Max	0.0040	0.0039	0.0044	0.0052	0.0055
Min	0.0072	0.0056	0.0088	0.0077	0.0100

**Table 7:** Procrustes distances between vertices of the allometrically scaled surfaces of heads at the maximum and minimum centroid sizes using the landmarks and highest density of semilandmarks and surfaces estimated using the landmarks and lower densities of semilandmarks after re-semilandmarking and re-warping.

	Size	20	40	60	80	100	200	400	600	800
Sliding TPS	Max	0.0075	0.0063	0.0066	0.0059	0.0056	0.0048	0.0031	0.0019	0.0014
	Min	0.0137	0.0102	0.0100	0.0091	0.0084	0.0083	0.0048	0.0031	0.0022
LS&ICP	Max	0.0140	0.0124	0.0120	0.0112	0.0103	0.0082	0.0050	0.0044	0.0030
	Min	0.0182	0.0160	0.0128	0.0145	0.0137	0.0104	0.0073	0.0067	0.0037
TPS&NICP	Max	0.0067	0.0049	0.0050	0.0041	0.0038	0.0036	0.0025	0.0019	0.0012
	Min	0.0119	0.0084	0.0079	0.0081	0.0083	0.0074	0.0056	0.0036	0.0017

**Table 8:** Procrustes distances between vertices of the estimated predictions of ape cranial surfaces at the maximum and minimum centroid sizes derived from the maximum density of semilandmarks and those from lower densities of semilandmarks after re-semilandmarking and re-warping.

	Size	50	100	200	400
Sliding TPS	Max	0.0139	0.0094	0.0076	0.0058
	Min	0.0267	0.0191	0.0146	0.0104
TPS&NICP	Max	0.0141	0.0099	0.0075	0.0058
	Min	0.0272	0.0199	0.0157	0.0119

## Data Availability

The data presented in this study are available upon request from the corresponding author.
